# Grapevines escaping trunk diseases in New Zealand vineyards have a distinct microbiome structure

**DOI:** 10.3389/fmicb.2023.1231832

**Published:** 2023-08-23

**Authors:** Damola O. Adejoro, E. Eirian Jones, Hayley J. Ridgway, Dion C. Mundy, Bhanupratap R. Vanga, Simon R. Bulman

**Affiliations:** ^1^Department of Pest-Management and Conservation, Faculty of Agriculture and Life Sciences, Lincoln University, Lincoln, Canterbury, New Zealand; ^2^The New Zealand Institute for Plant and Food Research Limited, Lincoln, Canterbury, New Zealand; ^3^The New Zealand Institute for Plant and Food Research Limited, Blenheim, Marlborough, New Zealand; ^4^Grapevine Improvement Laboratory, Bragato Research Institute, Lincoln, Canterbury, New Zealand

**Keywords:** *Vitis vinifera*, microbiome, *Pseudomonas*, grapevine trunk diseases, *Eutypa*, disease escape

## Abstract

Grapevine trunk diseases (GTDs) are a substantial challenge to viticulture, especially with a lack of available control measures. The lack of approved fungicides necessitates the exploration of alternative controls. One promising approach is the investigation of disease escape plants, which remain healthy under high disease pressure, likely due to their microbiome function. This study explored the microbiome of grapevines with the disease escape phenotype. DNA metabarcoding of the ribosomal internal transcribed spacer 1 (ITS1) and 16S ribosomal RNA gene was applied to trunk tissues of GTD escape and adjacent diseased vines. Our findings showed that the GTD escape vines had a significantly different microbiome compared with diseased vines. The GTD escape vines consistently harbored a higher relative abundance of the bacterial taxa *Pseudomonas* and *Hymenobacter*. Among fungi, *Aureobasidium* and *Rhodotorula* were differentially associated with GTD escape vines, while the GTD pathogen, *Eutypa*, was associated with the diseased vines. This is the first report of the link between the GTD escape phenotype and the grapevine microbiome.

## 1. Introduction

Plants are inhabited by a diverse assemblage of microorganisms (the microbiome), which influence all aspects of host productivity and health (Compant et al., 2019; Trivedi et al., 2020). The relationship of individual microorganisms with their plant hosts is categorized into beneficial, commensal, and pathogenic, and a key function of the plant microbiome is to protect against pathogens (McLaren and Callahan, 2020). The beneficial microorganisms may enhance the health of the plant by inducing innate plant defense systems, improving tolerance to stress conditions, promoting plant growth, and/or directly inhibiting pathogen growth by producing antimicrobial compounds (Araújo et al., 2002; Egamberdieva et al., 2017; Wang et al., 2017; Zeng et al., 2022; Mesguida et al., 2023). Moreover, the competition between commensals and invading pathogens could decrease the ability of the pathogens to enter and survive within the plant, thereby enhancing the plant’s resilience to diseases (McLaren and Callahan, 2020).

Recently, there has been an increased interest in studying the microbiomes of plants displaying a disease escape phenotype (Deyett et al., 2017; Kusstatscher et al., 2019; Ginnan et al., 2020). Disease escape describes a situation where plants remain healthy where they might otherwise be expected to show disease due to high local disease pressure (Deyett et al., 2017; Riera et al., 2017). Plants showing the disease escape phenotype are of interest because components of their microbiomes are believed to protect them against disease. For example, using amplicon sequencing, Deyett et al. (2017) aimed to understand the role of vascular endophytes of grapevines in Pierce’s disease escape phenotype. Their findings showed that two bacteria*, Pseudomonas fluorescens* and *Achromobacter xylosoxidans*, were inversely correlated with the presence of the pathogen, *Xylella fastidiosa*. *Pseudomonas fluorescens* was identified as a potential key player in the disease escape phenotype. Riera et al. (2017) identified bacterial strains from the rhizosphere of Huanglongbing (HLB) disease escape trees with antimicrobial properties against citrus pathogens. The study revealed that these bacterial strains, including *Burkholderia territorii, Burkholderia metallica, Pseudomonas geniculata*, and *Bacillus pumilus*, displayed antimicrobial properties against the surrogate bacterium for the HLB pathogen, *Candidatus* Liberibacter asiaticus, and the citrus root pathogen *Phytophthora nicotianae*. Similarly, Ginnan et al. (2020) identified key microbial taxa, such as *Lactobacillus* sp., *Exophiala* sp., *Aureobasidium* sp., and *Angustimassarina*, from HLB survivor (or escape) citrus trees. The researchers suggested that these taxa played a significant role in preserving the health of the HLB escape citrus trees. These findings indicate that harnessing the microbial components of disease escape plants could contribute to the development of innovative strategies for managing plant diseases and promoting plant health.

In recent years, the microbiome has been associated with the health status of humans, animals, and plants (Berendsen et al., 2012; Mueller and Sachs, 2015; Trivedi et al., 2020). The application of a microbiome-based approach for plant health improvement has been driven partly by the increasing recognition of the importance of the microbiome in human health and by the potential for manipulating the microbiome to improve plant growth and health (Compant et al., 2019; Bettenfeld et al., 2020, 2022; Mesguida et al., 2023) thus opening avenues for the control of chronic, complex, and intractable diseases, such as grapevine trunk diseases (GTDs). Grapevine trunk diseases are a diverse group of diseases caused by various fungi that invade and multiply within the perennial tissues of grapevines leading to a general deterioration of the vine’s woody structures (Mondello et al., 2018). As a result, the health of the grapevines progressively declines, as evidenced by internal wood necrosis, external wood cankers, delayed or lack of budburst from infected spurs, dieback of shoots and cordons, stunted growth, and chlorosis (Gramaje et al., 2018; Mondello et al., 2018). Grapevine trunk diseases cause significant yield losses, negatively impact vineyard productivity, and can ultimately lead to vine death (De La Fuente et al., 2016). Specific GTDs include esca, Botryosphaeria dieback, and Eutypa dieback. Among these, Botryosphaeria and Eutypa diebacks – which have a gradual onset and typically manifest symptoms in vines that are more than 8 years old – are the GTDs reported in New Zealand (Mundy and Manning, 2010). The pathology of GTDs is complex and evolving, with many aspects still under active research or knowledge gaps identified as opportunities for future research pursuits. Some of these include the identity and trophic modes of actions of the pathogens involved, the impact of abiotic stresses and nursery infections, the influence of grapevine genetics, the correlation between loss of vine vigor and eventual vine death, as well as the effectiveness and influence of management practices on vine health (Claverie et al., 2020). The complexity of GTDs is further underscored by the possibility for multiple fungal pathogens (with over 100 fungal species associated with GTD symptoms) to simultaneously infect grapevines (Gramaje et al., 2018). *Eutypa lata*, *Phaeomoniella chlamydospora*, and members of the Botryosphaeriaceae family have been widely studied as GTD pathogens. Moreover, the diseases progress slowly, and there is a potential for symptoms to overlap with other GTDs or abiotic stresses (Gramaje et al., 2018; Songy et al., 2019; Calvo-Garrido et al., 2021).

There are currently no approved fungicides for controlling GTDs, and this has contributed to the exploration of alternative approaches, such as a microbiome-based GTD management strategy, for controlling the diseases. The identification of the microbial composition of grapevines is not only one of the keys to devising effective management strategies but also a way of gaining a deeper understanding of GTDs. Consequently, researchers have characterized the microbiome of grapevine trunks from winegrowing regions worldwide. These studies have shown that the trunk mycobiome of healthy grapevines is usually composed of genera such as *Cladosporium, Aureobasidium, Alternaria, Epicoccum, Acremonium*, and *Phaeomoniella* (Bruez et al., 2016; Travadon et al., 2016; Del Frari et al., 2019; Niem et al., 2020). These fungi, along with GTD pathogens such as *E. lata, Phaeoacremonium, Diplodia, Neofusicoccum, Fomitiporia*, and *Diaporthe*, have been reported in GTD symptomatic grapevines (Niem et al., 2020; Bekris et al., 2021; Paolinelli et al., 2022). Similar fungi were reported from New Zealand, where *Phaeomoniella, Cladosporium, Eutypa, Epicoccum, Alternaria*, and *Aureobasidium* were shown to have the highest relative abundance in grapevine trunks (Vanga et al., 2022). It can be inferred from these studies that healthy and symptomatic grapevines share many fungal taxa. Many studies on the grapevine trunk microbiome have focused on the mycobiome, with the bacterial communities remaining relatively underexplored. Based on existing evidence from other plant microbiomes and limited studies on the grapevine trunk, it is likely that the grapevine trunk bacterial microbiome is predominantly composed of members from the Proteobacteria phylum (Turner et al., 2013; Lòpez-Fernàndez et al., 2017; Niem et al., 2020). For example, in Australian vineyards, Niem et al. (2020) found a high relative abundance of *Pseudomonas, Pedomicrobium, Hyphomicrobium, Jiangella*, and *Sphingomonas* in asymptomatic grapevine trunks. In contrast, Bekris et al. (2021) reported a high relative abundance of *Streptomyces, Bacillus, Acinetobacter*, and *Corynebacterium* from asymptomatic grapevine trunks in Greek vineyards. There is limited information in the literature to conclude whether the bacterial communities in healthy and symptomatic grapevine trunks are different.

There have been anecdotal reports of healthy vines persisting in backgrounds of heavy GTD pressure in New Zealand vineyards. Using surveys of four New Zealand vineyards, we have located candidate GTD escape vines (in prep). In this study, we applied a combination of DNA metabarcoding and microbial isolation to characterize the microbiomes of these plants and contrast them with diseased plants growing in the vicinity. The research was built around two pivotal questions: Do the microbiomes of candidate GTD escape vines differ from those of diseased vines? If so, are there microbial taxa in the GTD escape vines that can be linked to the observed GTD escape phenotype? We aimed to uncover microbial contributors to the expression of the GTD escape phenotype, which could help in the development of effective strategies for managing GTDs.

## 2. Materials and methods

### 2.1. Study regions and identification of grapevine trunk disease escape vines

We visited nine vineyards in Hawke’s Bay and Canterbury between November 2019 and February 2020 to identify candidate GTD escape vines. Hawke’s Bay and Canterbury, situated in North Island and South Island of New Zealand, respectively, are two of the major winegrowing regions in New Zealand. The vineyards in the Hawke’s Bay region have loamy soil, a mean air temperature of 13.2°C, and an annual rainfall of 716 mm (Manaaki Whenua – Landcare Research S-MAPONLINE: smap.landcareresearch.co.nz; NIWA National Climate Database^1^) (Chappell, 2013). Canterbury is a cooler region with a mean air temperature of 12.5°C. The Waipara Valley in Canterbury receives approximately 620 mm of rainfall annually (Macara, 2016), with the vineyards on silt-loam soils (smap.landcareresearch.co.nz). Candidate GTD escape vines in vineyards were differentiated from diseased vines using external symptoms of GTDs, such as leaf chlorosis, shoot stunting, poor canopy growth, and trunk cankers, as well as the chlorophyll content of grapevine leaves. Grapevines that did not have these GTD symptoms, despite being surrounded by vines showing the symptoms, were classified as GTD escape vines (see an example in Supplementary Figure 1). Following the identification of candidate GTD escape vines, grapevine trunk samples were collected from four vineyards, two each in the Hawke’s Bay (Fernhill/Ohiti/Ngatarawa subregion) and Canterbury (Waipara Valley) in February and March 2020.

### 2.2. Sample collection

A total of 73 woody trunk tissue samples, including 16 from candidate GTD escape vines and 57 from diseased vines nearby, were collected (Table 1) using the method of Mundy et al. (2018). A tissue sample from the trunk (approximately 80 cm above the soil) of each vine was collected by first removing the bark with a knife earlier disinfected with 70% ethanol. Woody tissue of the trunk was drilled to 40 mm using a 4-mm drill bit, sterilized in a 3% hypochlorite solution. About 1 g of trunk tissue sample was collected into 2 mL cryogenic tubes, frozen in liquid nitrogen, and kept at −80°C until DNA extraction. Samples for microbial isolations were kept cool on ice during transportation to the laboratory.

**Table 1:** Number of individual candidate GTD escape and diseased vines where grapevine woody trunk tissues samples were collected from the four vineyards used in the study.

Vineyard	Region	Number of candidate GTD escape vine	Number of diseased vine	Grape variety/rootstock	Year planted
Vineyard 1	Hawke’s Bay	5	19	Sauvignon blanc (SO4)	2005–2007
Vineyard 2	Hawke’s Bay	4	16	Sauvignon blanc (101–14)	2005–2006
Vineyard 3	Canterbury	4	13	Sauvignon blanc (own-rooted)	1985
Vineyard 4	Canterbury	3	9	Cabernet Sauvignon (own-rooted)	1987

### 2.3. DNA extraction

Genomic DNA was extracted from the woody trunk tissue samples using a cetyltrimethylammonium bromide (CTAB) method, as outlined in the study of Mundy et al. (2018). Blank DNA extractions were carried out during each run of DNA extraction. The DNA was quantified with a NanoDrop^®^ ND-1000 spectrophotometer, and its integrity was assessed by electrophoresis on 1% agarose gels. The gels were stained with SYBR Safe DNA gel stain (Thermo Fisher Scientific, Australia) and visualized under UV illumination using the Gel Doc EZ System (Biorad, United States).

### 2.4. Polymerase chain reaction amplification and sequencing

The fungal metabarcoding was as in Vanga et al. (2022), while the V5-V7 variable regions of the bacterial 16S rRNA gene were amplified with the primers 799F and 1193R (Chelius and Triplett, 2001; Bodenhausen et al., 2013). The primers were tagged with Illumina adapters (primer sequences are listed in Supplementary Table 1).

The DNA metabarcoding was performed in a two-step PCR. The first PCRs were performed in duplicate in a total volume of 20 μL containing 1 μL of template DNA, 10 μL of MyFi^™^ mix (Bioline), 1 μL of 10 μM of each primer, and 7 μL of molecular grade water. The PCR amplification was carried out in a T100 thermal cycler (Bio-Rad) with the following program: initial denaturation at 94°C for 3 min, 35 cycles (30 cycles for bacteria) of denaturation at 94°C for 30 s, annealing at 55°C for 30 s, elongation at 72°C for 60 s and a final extension phase at 72°C for 5 min, before holding at 12°C. All PCRs included a negative, no template control. The mock DNA community, ZymoBIOMICS microbial standard (Zymo Research, Irvine, CA, United States), was included as a positive control. All PCR products were visualized in 1% agarose (at 100 V for 30 min). The PCR products were purified using Agencourt^®^ AMPure^®^ XP magnetic beads (Beckman Coulter). Sample-specific barcodes were added to the purified amplicons in a second PCR reaction consisting of 10 μL MyFi^™^ mix (Bioline), 1 μL each of 5 μM barcoding primers, 1 μL template from the first round of PCR, and 7 μL molecular grade water (20 μL total). The PCR reaction cycle was 95°C for 3 min; five cycles of 95°C for 20 s, 55°C for 15 s, and 72°C for 30 s; and a final 72°C extension for 60 s before holding at 12°C. The indexed PCR products were purified with Agencourt^®^ AMPure^®^ XP magnetic beads and quantified with a NanoDrop^®^ ND-1000 spectrophotometer. Amplicons were pooled equimolarly and sequenced on the Illumina MiSeq platform using 2 × 300 bp paired-end reads with the MiSeq reagent kit v3 chemistry (Auckland Genomics, New Zealand).

### 2.5. Bioinformatics

Demultiplexed raw reads obtained from Auckland Genomics were quality-filtered, denoised, chimera-checked, and processed into amplicon sequence variants (ASV) using the DADA2 (v1.16.0) R package (Callahan et al., 2016). The ITS primer sequences were trimmed with cutadapt v1.3.1 without further truncation. The 16S rRNA primer sequences were removed using the *trimLeft* function and truncated at 260 bp for the forward and reverse reads. The taxonomic assignment of the ASVs was conducted using the *assignTaxonomy* function in DADA2. The UNITE (10.05.2021) database (Nilsson et al., 2019) and the SILVA (v138.1) rRNA database (Quast et al., 2012) were used as reference databases for fungi and bacteria, respectively. Sequences belonging to mitochondria, chloroplast, and ASVs not classified at the kingdom and phylum levels were removed from the dataset. The ASV counts data was decontaminated using microDecon (McKnight et al., 2019). For further downstream analysis, files were either used as inputs for MicrobiomeAnalyst (Chong et al., 2020) or made into a phyloseq object and analyzed with the phyloseq R package (McMurdie and Holmes, 2013) on R (v. 4.2.0) (R Core Team, 2013).

#### 2.5.1. Sequence data analysis

The relative abundance of fungal and bacterial genera was calculated with phyloseq after normalizing the ASV counts with the cumulative sum scaling method (Paulson et al., 2013). The taxonomic composition of grapevine trunk microbiomes was visualized with GraphPad Prism v 8.4.3 (GraphPad Software, California, United States). Statistical analysis was completed on pooled samples across vineyards.

Alpha diversity was calculated with phyloseq, using data scaled by ranked subsampling (Beule and Karlovsky, 2020). The statistical significance of alpha diversity values was then calculated using the Wilcoxon rank sum test (*p* < 0.05). The results were visualized with ggplot2. The principal coordinate analysis (PCoA) using the Bray-Curtis distance method was carried out with the *ordinate* function, and the results were visualized with the *plot_ordination* function in the phyloseq package. The permutational multivariate analysis of variance (PERMANOVA) test with 999 permutations was used to analyze the statistical effect of the grapevine condition on the fungal and bacterial community structures of GTD escape and diseased vines, using the *adonis* function from the vegan package within the R software environment (Oksanen, 2010).

The discriminating ASVs between GTD escape and diseased vines were identified with edgeR (Robinson et al., 2010) and the DESeq2 R packages (Love et al., 2014), as recommended by McMurdie and Holmes (2014). The differential abundance analysis pipeline outlined by Xia et al. (2018) was followed. The edgeR results were visualized by a volcanic plot with label texts positioned with the ggrepel R package (Slowikowski, 2018). Further identification of taxa shaping the grapevine trunk microbiome was performed using a Random Forest model (Breiman, 2001), as implemented in the randomForest package (Liaw and Wiener, 2002) within MicrobiomeAnalyst (Chong et al., 2020). The Random Forest model was trained with 5000 trees and a randomness setting of 123456, with the results visualized in GraphPad Prism.

The pattern search tool on MicrobiomeAnalyst, using the SparCC algorithm (Friedman and Alm, 2012), was used to identify fungal genera correlated with *Eutypa*. Network analysis of the GTD escape and diseased samples was carried out with the ggClusterNet R package (Wen et al., 2022) based on Pearson’s correlations. Strong (*r* > 0.60) and statistically significant (*p*-value < 0.05) Pearson’s correlations were accepted. Only ASVs with a mean relative abundance >0.01% were used for the network construction. Within kingdom analysis was conducted with the *network.2* function, while bipartite network analysis between fungi and bacteria was conducted with the *corBionetwork* function. The networks were visualized with the *model.maptree2* function. The topological roles of taxa were revealed by calculating the within-module connectivity (Zi) and among-module connectivity (Pi). Taxa that belong to the connector (Zi ≤ 2.5, Pi≥0.62), module (Zi ≥ 2.5, Pi ≤ 0.62), and network hubs (Zi ≥ 2.5, Pi ≥ 0.62) (Olesen et al., 2007) can be considered keystone taxa (Yuan et al., 2021).

### 2.6. Isolation and identification of microorganisms from grapevine trunk

Three pieces of tissue from each grapevine wood sample, between 2 and 5 mm, were placed onto potato dextrose agar (PDA, Difco^™^, United States) and malt extract agar (MEA, Difco^™^, United States), amended with 50 mg/L of streptomycin for the isolation of fungi. For the selective isolation of basidiomycetes, separate MEA plates were further amended with benomyl (4 mg/L) to retard the growth of ascomycetes (Bonito et al., 2016). Plates were incubated at 25°C in the dark. The plates were observed regularly for fungal colonies growing from the tissue pieces and these were sub-cultured onto fresh agar as needed for up to 3 weeks. The fungal cultures were further sub-cultured until pure cultures were obtained. Isolates were stored as mycelial agar plugs in 30% glycerol at −80°C. Fungal isolates with similar colony morphology (surface and reverse appearances on plates) were grouped into morphotypes. A representative isolate from each morphotype was grown for 10 days on PDA, Sabouraud dextrose agar (SDA, Oxoid, United Kingdom), MEA, Czapek Dox agar (CDA, Oxoid, United Kingdom), and 2% water agar, in the dark, at 25°C for capturing photographs of colony morphotypes. For bacterial isolation, three grapevine wood tissue pieces were plated on triplicate R2A agar (Difco^™^, United States) plates amended with cycloheximide at 40 μg/mL to retard fungal growth (Gdanetz and Trail, 2017). The plates were incubated at 25°C in the dark and checked every 2–3 days for 4 weeks with any bacterial colonies sub-cultured onto fresh agar. Purified bacterial isolates were grown on nutrient agar (Oxoid, United Kingdom) for 2–3 days before DNA extraction.

Genomic DNA was extracted from the fungal mycelia and single colonies of pure bacterial cultures using the Extract-N-Amp^™^ Plant PCR Kit (Sigma Aldrich). The ITS region of fungal isolates was amplified by PCR with the primers ITS5 and ITS26 (Khan et al., 2013), while the 16S rRNA gene region of all bacterial isolates was amplified using the universal primer pair of 27F and 1492R (Weisburg et al., 1991). The PCR products were purified using the Agencourt^®^ AMPure^®^ XP magnetic beads (Beckman Coulter, USA) and directly Sanger-sequenced at Macrogen Inc. (Seoul, South Korea). The raw sequences obtained from Macrogen Inc. were checked and cleaned in Geneious Prime (v2022.0.1). Taxonomic assignment of the isolates was performed by aligning their sequences with the NCBI ‘nt’ database using the BLAST algorithm (Altschul et al., 1997). The best match was selected after considering the *e*-value and the percentage of sequence identity. In order to compare the *Pseudomonas* isolates with the *Pseudomonas* ASVs, their sequences were aligned with selected reference *Pseudomonas* sequences from the NCBI ‘nt’ database using the MUSCLE algorithm (Edgar, 2004) in MEGA 11, with the default parameters (Tamura et al., 2021). All resulting alignments were trimmed to equal lengths and were visually inspected for accuracy. Neighbor-joining trees (Saitou and Nei, 1987) were constructed in MEGA 11.

## 3. Results

### 3.1. Sequence data summary

Three woody trunk tissue samples (one diseased sample from vineyard 1 and two from vineyard 2) were unusable due to DNA extraction failure. The final number of wood samples processed for DNA sequencing was 70 (16 candidate GTD escape vine samples and 54 diseased vine samples). From the fungal dataset, 1,431,513 reads remained out of 3,632,192 raw reads after filtering and chimera removal. For the bacterial dataset, 2,856,153 reads remained out of 5,271,359 raw paired reads after the filtering and chimera removal steps in DADA2. Only samples with at least 1,000 reads were retained for further downstream analyses. Because of this, three and six samples were eliminated from the fungal and bacterial datasets, respectively. Overall, 754 ASVs were identified from 68 samples in the fungal dataset, while 3,716 ASVs were identified from 65 samples in the bacterial dataset. Twelve fungal ASVs, including *Malassezia, Cryptococcus*, and *Ramularia*, and 20 bacterial ASVs, including *Cutibacterium, Micrococcus*, and *Streptococcus*, were identified as contaminants by microdecon and removed from the dataset (Supplementary Data 1).

### 3.2. Taxonomic composition of samples

#### 3.2.1. Taxonomic composition of the fungal community

**Figure 1:**
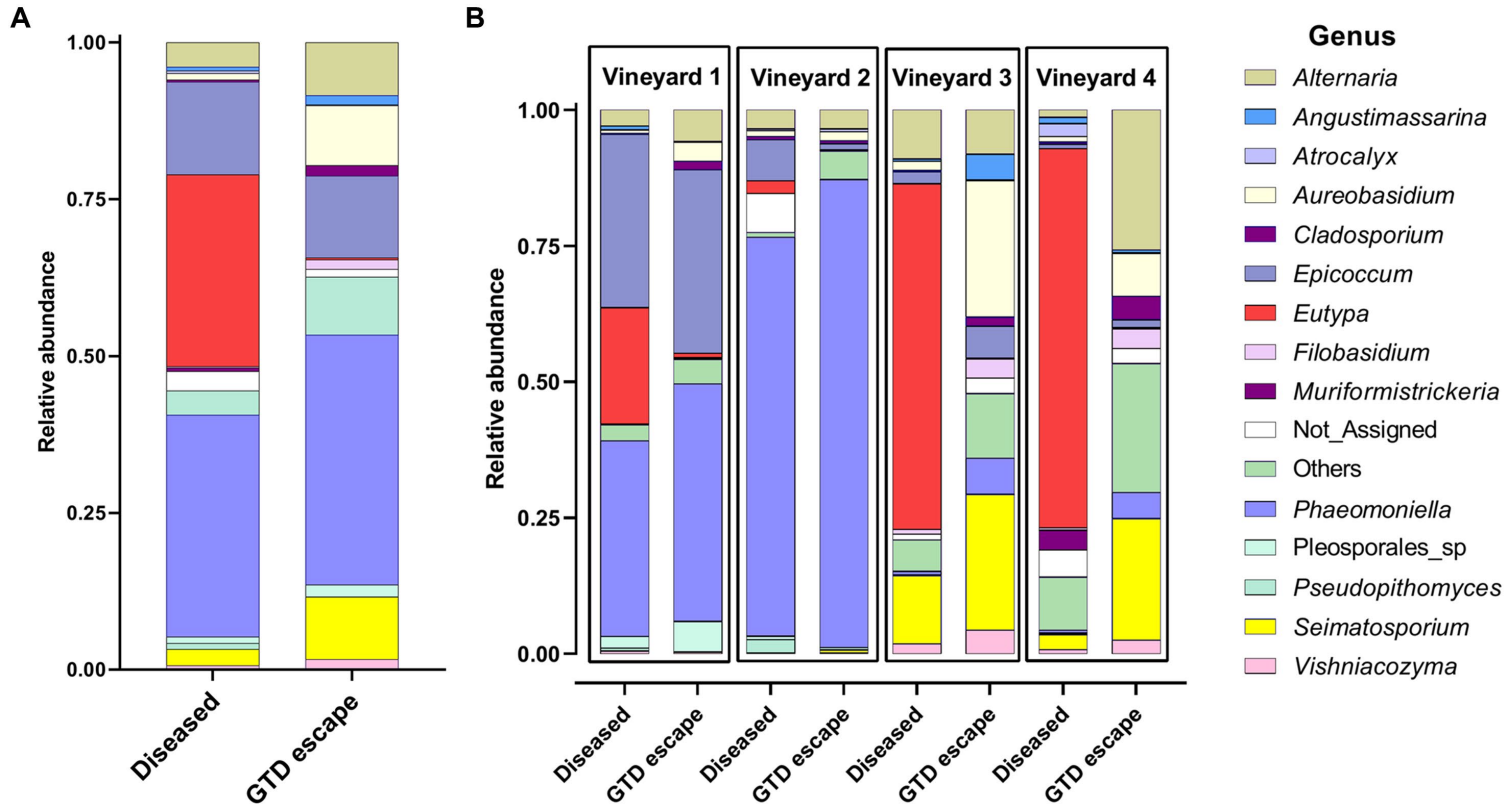
The relative abundance of the 14 relatively most abundant fungal genera in the woody trunk tissue of all diseased and candidate GTD escape vine samples **(A)** and in the woody trunk tissue of diseased and candidate GTD escape vine samples in individual vineyards **(B)**. ‘Not_Assigned’ are taxa not identified at the genus level, while ‘Others’ are taxa not among the 14 most abundant taxa.

Overall, the fungi were assigned to 76 genera. Among the GTD escape vines, *Phaeomoniella* (40%), *Epicoccum* (13%), *Seimatosporium* (10%), *Aureobasidium* (10%), and *Alternaria* (8%) had the highest relative abundance (Figure 1A). In the diseased samples, *Phaeomoniella* (35%), *Eutypa* (31%), *Epicoccum* (15%), *Alternaria* (4%), and *Seimatosporium* (3%) were the genera with the highest relative abundance. Among these fungal genera, *Eutypa* (GTD escape 0.3% – diseased 31%), *Aureobasidium* (GTD escape 10% – diseased 1%), *Seimatosporium* (GTD escape 10% – diseased 3%) and *Cladosporium* (GTD escape 2% – diseased 0.4%) showed the highest variation in relative abundance between the GTD escape and diseased vines. Many of the substantial differences in the relative abundance of these fungi between GTD escape and diseased vines were consistent across all the vineyards (Figure 1B). The high relative abundance of *Phaeomoniella* was primarily observed in vineyards located in the Hawke’s Bay, but notably lower in the Canterbury vineyards. The relative abundance of *Eutypa* was increased in all four vineyards for diseased samples, although with a low relative abundance in vineyard 2. *Eutypa* also had a higher relative abundance in the diseased samples from the Canterbury vineyards than the diseased samples from Hawke’s Bay. Other GTD pathogens detected include *Neofusicoccum*, *Diplodia*, *Diaporthe*, and *Phaeoacremonium*, but these were at low relative abundance (<0.4%).

#### 3.2.2. Taxonomic composition of the bacterial community

**Figure 2:**
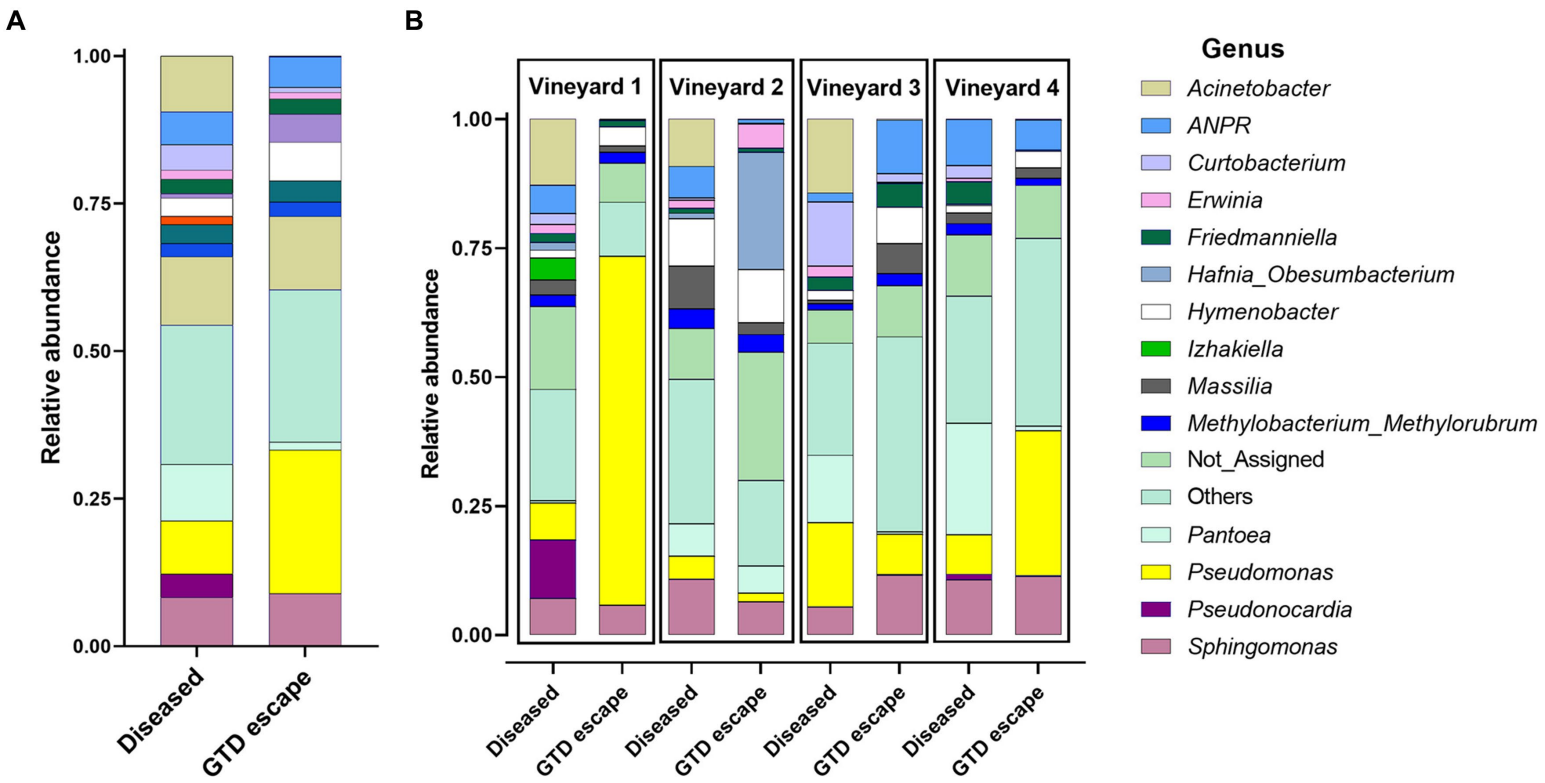
The relative abundance of the 14 relatively most abundant bacterial genera in the woody trunk tissue of all diseased and candidate GTD escape vine samples **(A)** and in the woody trunk tissue of diseased and candidate GTD escape vine samples in individual vineyards **(B)**. ANPR is *Allorhizobium-Neorhizobium-Pararhizobium-Rhizobium*, ‘Not_Assigned’ are taxa not identified at the genus level, while ‘Others’ are taxa not among the 14 most abundant taxa.

**Figure 3:**
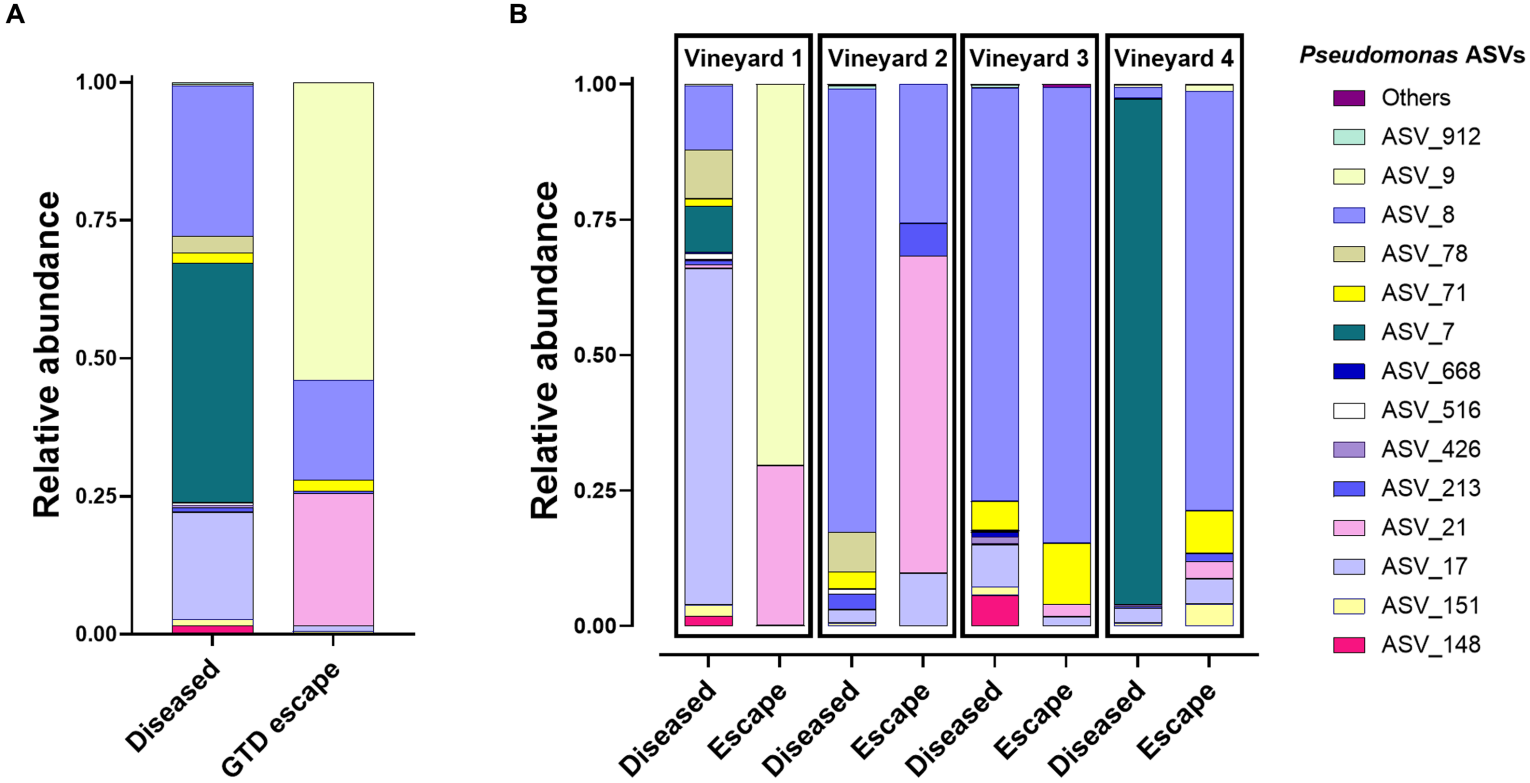
Distribution of the 14 *Pseudomonas* ASVs with the highest relative abundance in GTD escape and diseased vines **(A)** and across vineyards **(B)**. The relative abundance of these *Pseudomonas* ASVs has been corrected to make them equal to 100%.

Most bacteria belonged to the phylum Proteobacteria (GTD escape 69% – diseased 70%). Other phyla represented were Actinobacteriota (GTD escape 14% – diseased 20%), and Bacteroidota (GTD escape 15% – diseased 20%). Firmicutes had a lower representation with 1.5% ASVs in diseased vines and less than 1% ASVs in GTD escape vines. Overall, the bacteria were assigned to 252 genera. *Pseudomonas* (24%) had the highest relative abundance in GTD escape vines, followed by *Sphingomonas* (9%), *Hymenobacter* (7%), *Allorhizobium-Neorhizobium-Pararhizobium-Rhizobium* (*ANPR*) (5%) and *Hafnia_Obesumbacterium* (5%) (Figure 2A). The taxa with the highest relative abundance in diseased vines were *Pantoea* (10%), *Acinetobacter* (9%), *Pseudomonas* (9%), *Sphingomonas* (8%), and *ANPR* (6%). The higher relative abundance of *Hymenobacter* in the GTD escape vines was consistent across all the vineyards, while the trend for *Pseudomonas* was observable only in vineyards 1 and 4. *Pantoea* and *Curtobacterium* also had a higher relative abundance in diseased vines across all four vineyards (Figure 2B). Due to the variable pattern of *Pseudomonas*’ increased relative abundance in GTD escape vines across the vineyards, we investigated the distribution of all ASVs classified as *Pseudomonas* in the vineyards (Figures 3A,B). The relative abundance of ASV 9 was higher in GTD escape vines than in diseased vines in vineyard 1 (Figure 3B). The highest relative abundance of ASV 21 was observed in the GTD escape vines in vineyard 2, and the ASV was relatively higher in GTD escape vines than in diseased vines in the other three vineyards. In vineyard 4, *Pseudomonas* ASV 7 dominated the diseased samples.

#### 3.2.3. Alpha diversity

The GTD escape vines had significantly higher fungal ASV richness than the diseased vines (*p* < 0.02), but the Shannon and Simpson diversity indexes were similar between the two conditions (Supplementary Figure 2). There were no significant differences in the diversity and community richness between the bacterial ASVs in GTD escape and diseased vines (Supplementary Figure 3).

#### 3.2.4. Beta diversity

**Figure 4:**
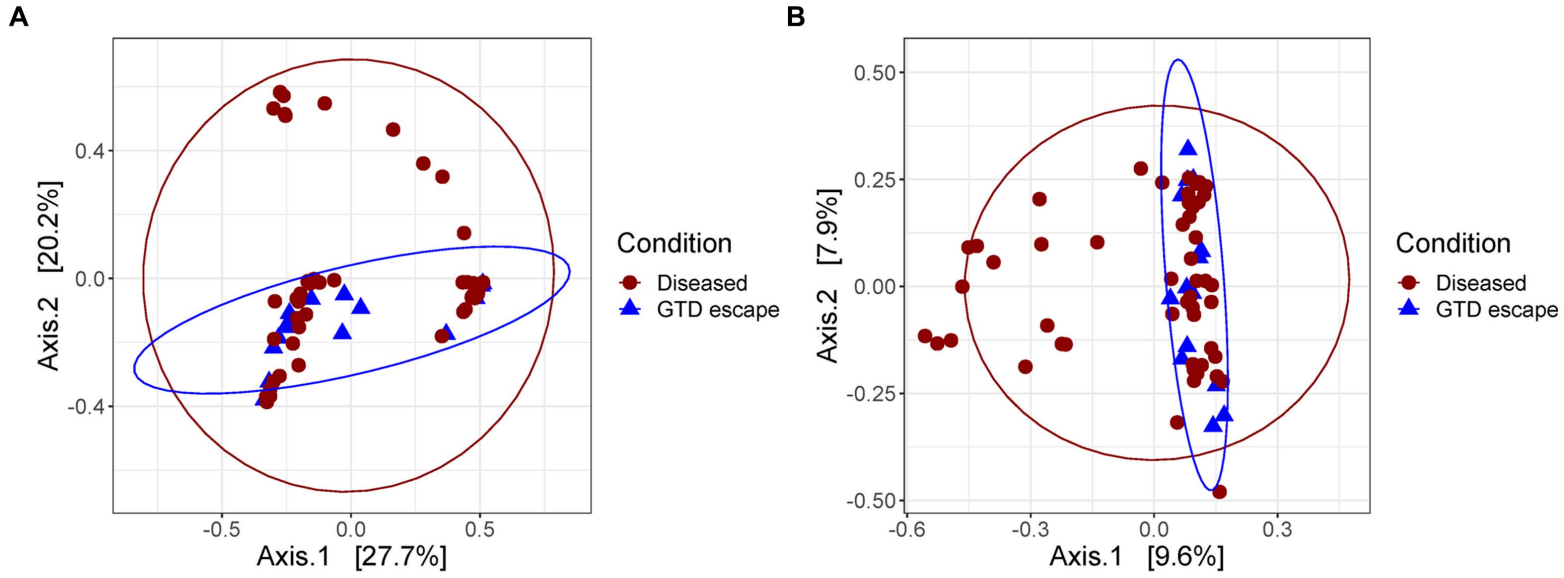
Principal coordinate analysis plot based on the Bray–Curtis dissimilarity index showing the distances between the fungal **(A)** and bacteria **(B)** communities of diseased and GTD escape vines. Each colored shape represents an individual sample.

The Bray-Curtis clustering of both fungal and bacterial communities demonstrated that GTD escape vines grouped together (Figures 4A,B). Further statistical analysis using PERMANOVA corroborated this observation. Whether vines were classified as GTD escape or diseased significantly influenced the fungal (*p* = 0.043) and bacterial (*p* = 0.008) communities, explaining 2.99 and 2.47% of the variation observed in the fungal and bacterial community structures, respectively.

### 3.3. Differential abundance analysis

**Figure 5:**
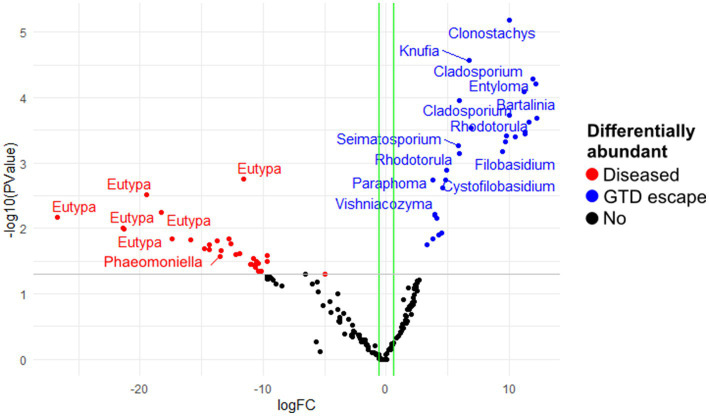
The differentially abundant fungal amplicon sequence variants (ASV) between diseased and GTD escape vine trunk samples, as identified by edgeR. The red and blue points are the ASVs that showed large-fold changes and high statistical significance (*p* < 0.05) in the diseased and candidate GTD escape samples, respectively. The black points are ASVs that were not differentially abundant in the two groups. The gray line is the y-intercept of -log10 (*p*-value).

The analysis of fungal and bacterial communities in diseased and GTD escape vines using edgeR revealed that fungal genera, including *Cladosporium, Seimatosporium, Rhodotorula*, and *Clonostachys*, were statistically associated with GTD escape vines, while *Eutypa* was the major genus that was associated with diseased vines (Figure 5). Other fungal taxa statistically associated with the diseased vines were *Ramularia*, *Diaporthe*, *Phaeomoniella, Diplodia,* and *Neofusicoccum* (Supplementary Data 1). The DESeq2 analysis revealed four ASVs of *Eutypa* and one of *Alternaria* to be differentially abundant in diseased samples. No fungal ASV showed such differential abundance in GTD escape samples, according to DESeq2.

**Figure 6:**
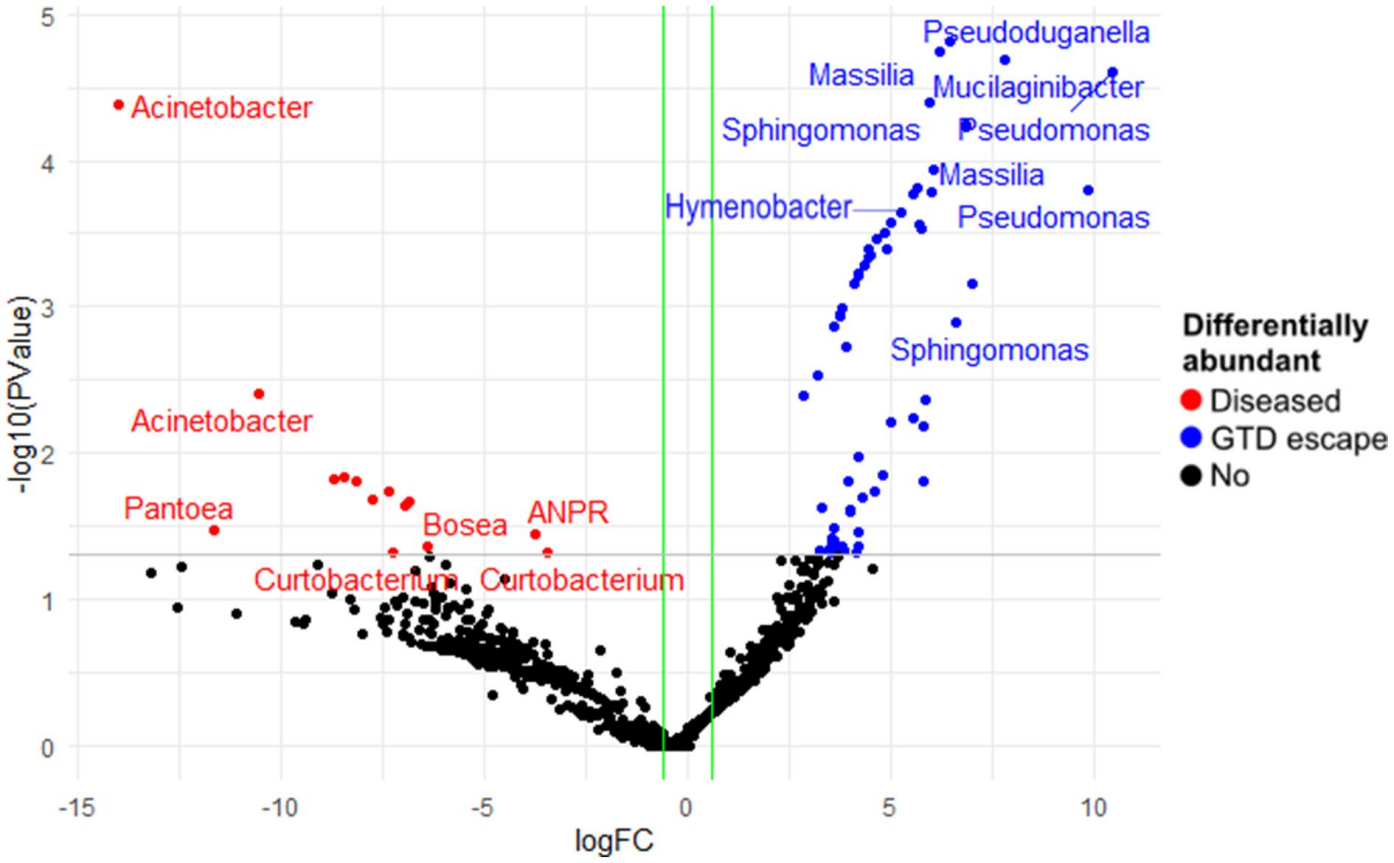
The differentially abundant bacterial amplicon sequence variants (ASV) between diseased and GTD escape vine trunk samples, as identified by edgeR. The red and blue points are the ASVs that showed large-fold changes and high statistical significance (*p* < 0.05) in the diseased and candidate GTD escape samples, respectively. The black points are ASVs that were not differentially abundant in the two groups. The grey line is the y-intercept of -log10 (*p*-value).

In the bacterial community, edgeR showed the highest differential abundance in the GTD escape vines was from the genera *Pseudomonas, Massilia, Mucilaginibacter, Sphingomonas*, and *Hymenobacter* (Figure 6). *Acinetobacter* and *Pantoea* were shown by edgeR and DESeq2 as differentially abundant taxa in diseased vines (Figure 6 and Supplementary Data 1). Only one differentially abundant bacterial ASV (*Pseudomonas* ASV 21) was identified by DESeq2 in the GTD escape vines. In contrast a *Pseudomonas* ASV 7, which was prominent in vineyard 4, was associated with the diseased vines.

### 3.4. Random Forest analysis of microbial communities

**Figure 7:**
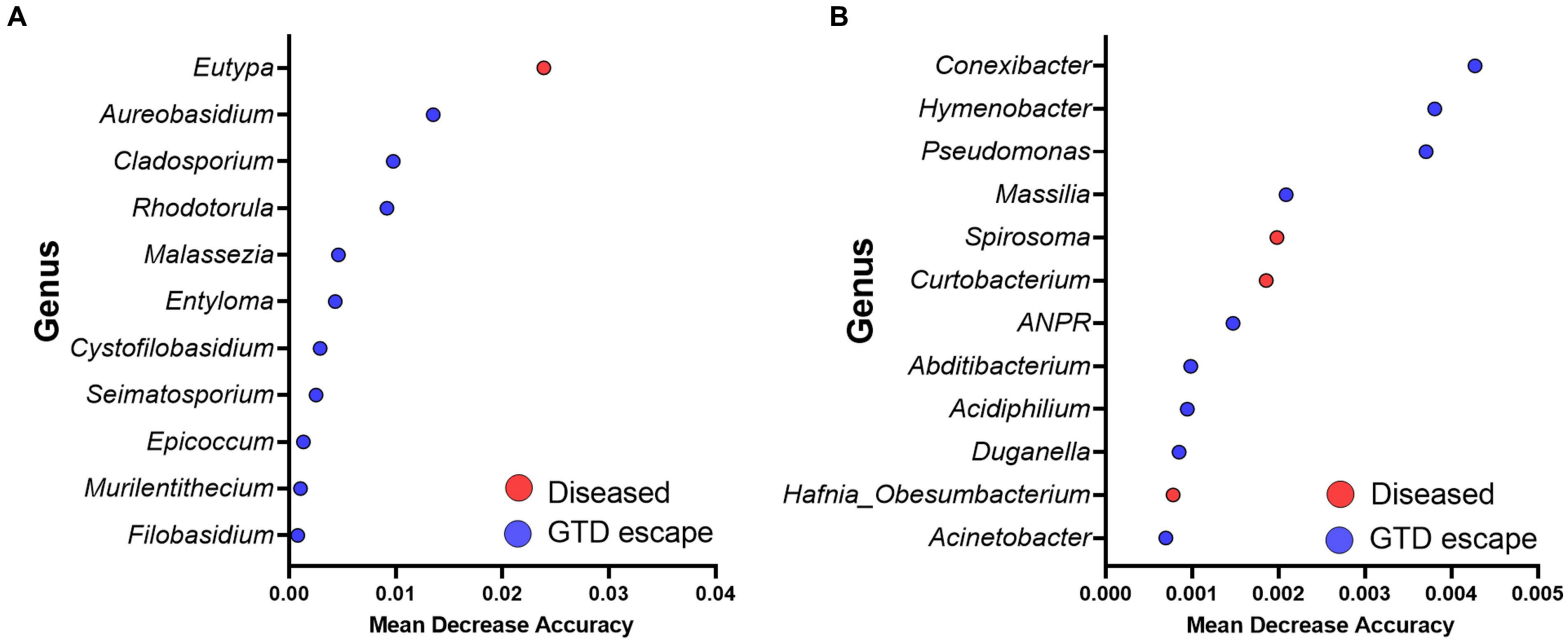
Summary of the significant genera driving the fungal **(A)** and bacterial **(B)** communities revealed by the Random Forest analysis. Red and blue dots show taxa with significantly higher abundance in diseased and GTD escape vines, respectively. The mean decrease accuracy measures the importance of each taxon to the Random Forest model.

The Random Forest model showed that *Eutypa* was the major genus responsible for the fungal structural differences observed in the trunk microbiome of diseased vines. However, in the GTD escape vines, the fungal community structure was driven by fungal genera such as *Aureobasidium, Cladosporium, Rhodotorula*, and *Seimatosporium* (Figure 7A). The bacterial microbiome of the GTD escape samples was influenced by *Hymenobacter, Pseudomonas*, and a low abundance genus, *Conexibacter*. In contrast, the microbiome of diseased vines was significantly influenced by *Spirosoma*, *Curtobacterium*, and *Hafnia_Obesumbacterium* (Figure 7B).

### 3.5. Correlation and network analyses

**Figure 8:**
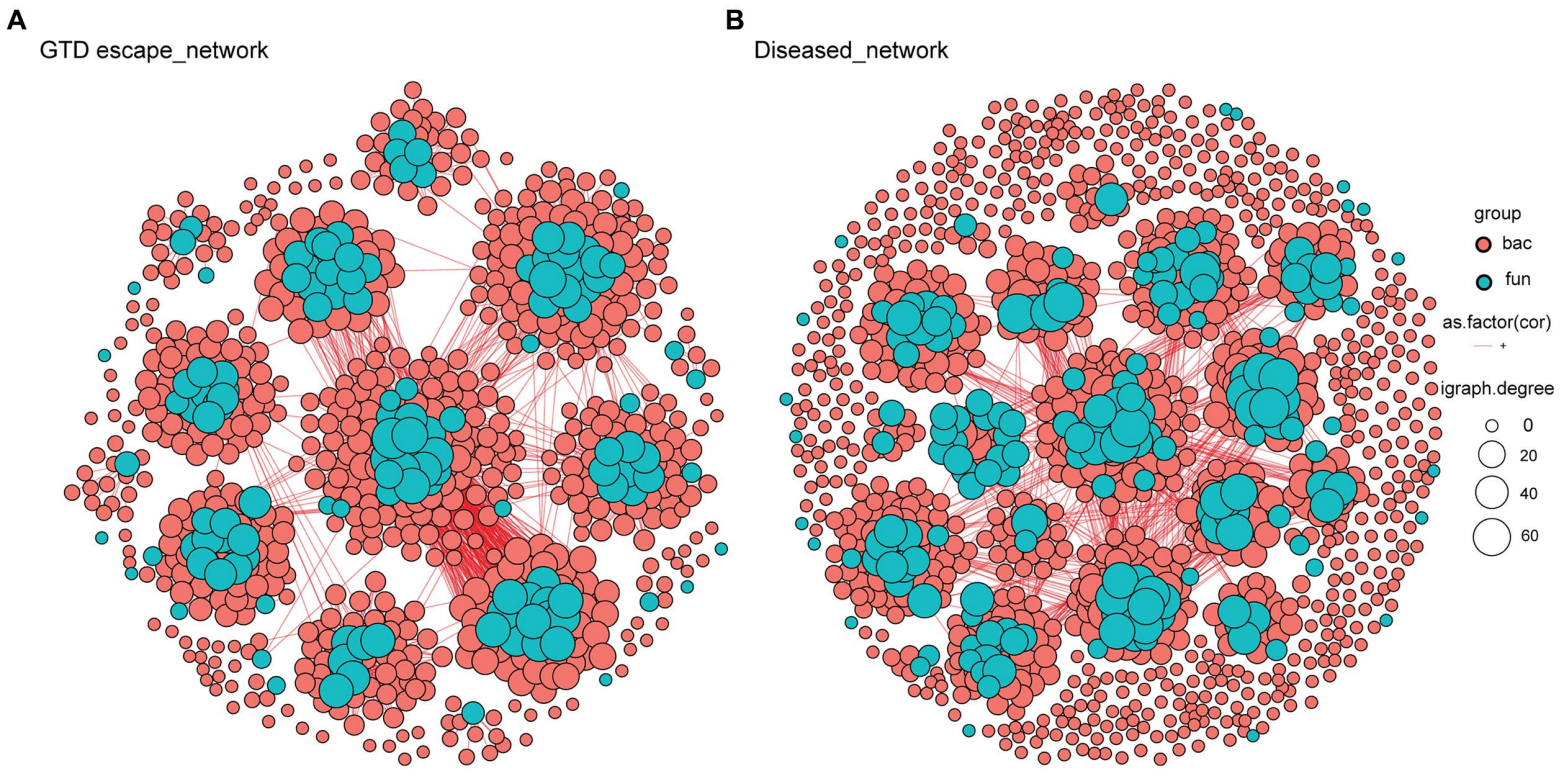
Bipartite networks of GTD escape **(A)** and diseased **(B)** vines based on Pearson’s correlation analysis. Each node, colored by its kingdom (bacteria – pink, fungi – blue), represents an ASV. Their size is proportional to the degree of the individual ASV. Connections between nodes signify strong (*r* > 0.6) and statistically significant (*p* < 0.05) correlations.

The analysis of taxa that correlated with *Eutypa* showed that relatively highly abundant genera, including *Cladosporium, Epicoccum, Aureobasidium, Vishniacozyma*, and *Rhodotorula* were significantly negatively correlated with *Eutypa* (Supplementary Data 2). In contrast, the genera that correlated positively with *Eutypa*, such as *Devriesia, Ustilago*, and *Bensingtonia*, were all very low in relative abundance. Microbial network complexity and stability were measured using diameter, nodes, clusters, edges, average path length, and centralization betweenness (Table 2 and Supplementary Datas 3–5). The complexity of the bipartite network reduced in the presence of GTDs. This was indicated by a decrease in the number of network nodes and edges from 651 and 4089, respectively, in the GTD escape network to 639 nodes and 2093 edges in the diseased network (Table 2 and Figure 8). The individual GTD escape networks for fungi and bacteria had more edges but fewer nodes than the diseased network (Table 2 and Supplementary File 1). The GTD escape networks were also more stable than diseased networks, as indicated by their higher relative modularity (Table 2).

**Table 2:** General characteristics of GTD escape and diseased networks in fungi, bacteria and bipartite analyses.

Network feature	Fungi		Bacteria		Bipartite (fungi and bacteria)	
	GTD escape	Diseased	GTD escape	Diseased	GTD escape	Diseased
Number of nodes	137	177	586	901	651	639
Number of edges	940	794	12522	8531	4089	2093
Diameter	6.23	6.49	7.55	21.57	13.00	14.00
Number of clusters	11	20	4	11	7	9
Relative modularity	2.80	2.10	8.19	4.87	NA	NA
Average path length	2.09	2.89	3.04	7.74	5.09	5.48
Average degree	13.72	8.97	42.74	18.94	12.56	6.55
Centralization betweenness	0.10	0.10	0.15	0.18	0.14	0.08
Centralization degree	0.17	0.10	0.06	0.02	0.10	0.04
Centralization closeness	0.65	0.92	1.42	1.57	1.53	1.55

An ASV belonging to the *Boeremia* genus was the only potential keystone fungal taxon detected in the GTD escape network (Supplementary Data 6). However, six putative keystone bacterial taxa, namely *Segibacter*, *Actinoplanes*, *Roseomonas*, a Sphingomonadaceae, *Mucilaginibacter*, and *Massilia*, were detected within the GTD escape network (Supplementary Data 6). Interestingly, different ASVs belonging to *Mucilaginibacter* and *Massilia* were also detected as possible keystone taxa in the diseased network. *Mucilaginibacter* and *Massilia* were module and connector hubs in the networks, respectively.

### 3.6. Fungi and bacteria isolated from grapevine trunks

**Figure 9:**
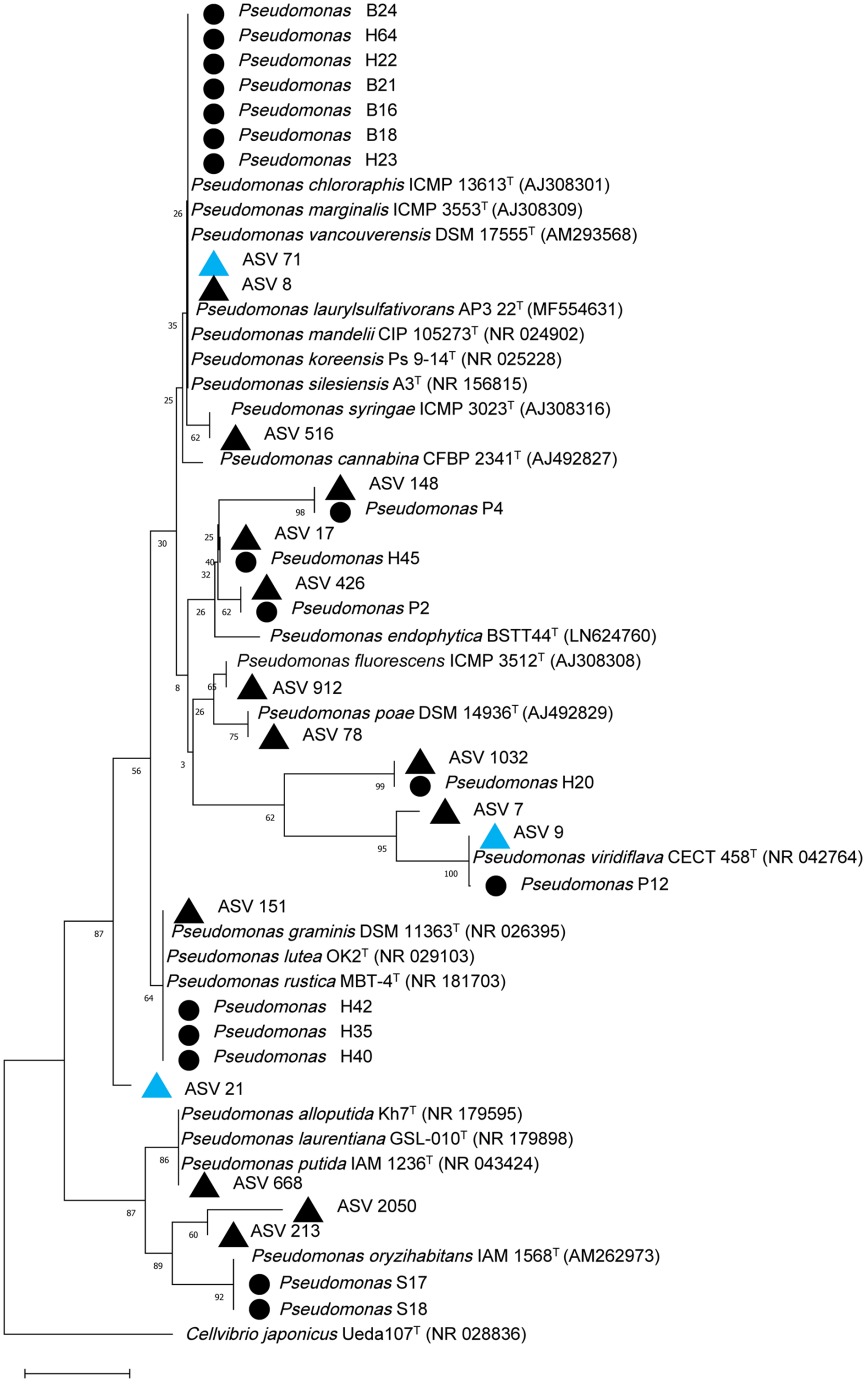
Phylogenetic relationship based on partial 16S rRNA gene sequences (~300 bp) of 18 *Pseudomonas* isolates and top *Pseudomonas* ASVs in the grapevine endosphere with closely related sequences, known pathogenic and beneficial *Pseudomonas* and an outgroup sequence of *Cellvibrio japonicus* using the Neighbor-joining method in MEGA 11. The numbers at the tree nodes are bootstrap values based on 1,500 bootstrap replicates. The bar (0.01) represents the number of mutations per sequence position. *Pseudomonas* isolates from this study are designated with a black circle, while *Pseudomonas* ASVs with higher relative abundance in GTD escape or diseased vines are designated with blue or black triangles, respectively.

A total of 1,343 fungal isolates (631 from candidate GTD escape vines and 712 from diseased vines) were obtained. The fungal isolates were placed into 20 morphotypes (Supplementary Figures 4–6). Following molecular identification, the main fungal genera were *Epicoccum, Cladosporium, Fusarium, Eutypa, Botryosphaeria, Diplodia, Mucor, Alternaria, Aureobasidium, Seimatosporium*, and *Penicillium* (Supplementary Table 2). For the bacteria, 151 isolates were obtained (84 from GTD escape vines and 67 from diseased vines). The most commonly isolated bacterial genera were *Pseudomonas* (18 isolates), *Pantoea* (17 isolates), *Curtobacterium* (15 isolates), and *Erwinia* (9 isolates) (Supplementary Table 3). The phylogenetic analyses of the 18 *Pseudomonas* isolates and the *Pseudomonas* ASVs showed considerable sequence variability (Figure 9). Some isolates clustered within the same group containing *P. fluorescens*, while three isolates and one ASV clustered in the same group with *P. lutea*. A *Pseudomonas* isolate and ASV 9, which had higher relative abundance in GTD escape than in diseased vines, clustered with *P. viridiflava*. Similarly, ASV 7, which had a high relative abundance in diseased vines, grouped with *P. viridiflava*.

## 4. Discussion

In this study, we applied high-throughput sequencing methods to investigate the microbial community structure of grapevines that escape GTDs. Utilizing DNA metabarcoding, we examined the fungal and bacterial community structures within the trunk endosphere of candidate GTD escape vines and compared them with nearby diseased vines. The findings revealed significant variations in the microbiome structure between the GTD escape and diseased vines, with specific microbial taxa driving these distinctions. These microbial taxa may contribute to the expression of the GTD escape phenotype.

The key fungal driver of the microbiome difference between GTD escape and diseased vines was *Eutypa*. This fungus showed a significant increase in relative abundance in diseased samples and was consistently discriminant for the samples. *Eutypa lata* is a known GTD pathogen (Carter, 1991), and the consistent differences observed with *Eutypa* between GTD escape and diseased vines across all the vineyards support previous observations that *E. lata* is a major GTD pathogen in New Zealand (Mundy et al., 2020; Vanga et al., 2022). While *Eutypa* was relatively more abundant in diseased than GTD escape samples, the scale of its presence and its role in the symptoms of GTDs observed in vineyard 2 may warrant further investigation. The consistently higher relative abundance of *Eutypa* in diseased vines showed that our identification of these vines was accurate despite the known latency of GTD pathogens such as *Phaeomoniella* (Hrycan et al., 2020) and the annual fluctuation in the expression of Eutypa dieback foliar symptoms (Sosnowski et al., 2007). Other GTD pathogens reported elsewhere, such as the Botryosphaeriaceae, *Phaeoacremonium*, and *Fomitiporia*, were either not detected or detected at low relative abundance in this study. These fungi are not widespread GTD pathogens in New Zealand (Vanga et al., 2022). The other fungi found in this study, such as *Phaeomoniella, Epicoccum, Seimatosporium, Cladosporium, Aureobasidium*, and *Alternaria*, have been frequently found in close association with grapevines worldwide (González and Tello, 2011; Del Frari et al., 2019; Bekris et al., 2021; Liu and Howell, 2021). Furthermore, the mycobiome of the vines in this study was similar to those reported in recent grapevine trunk studies in New Zealand (Vanga et al., 2022) and overseas (Niem et al., 2020; Bekris et al., 2021). For example, Vanga et al. (2022) reported that *Phaeomoniella, Cladosporium*, *Eutypa, Epicoccum, Alternaria,* and *Aureobasidium* had the highest relative abundance in the trunks of grapevines from vineyards in Marlborough, New Zealand. Similarly, Niem et al. (2020) found *Phaeomoniella*, *Phaeoacremonium*, and *Alternaria* associated with grapevines in Australian vineyards. Additionally, the routine isolation of these fungi aligns with other grapevine studies (González and Tello, 2011; Bruez et al., 2016; Kraus et al., 2019). *Trichoderma*, frequently employed as a biological control agent for managing GTDs (Mesguida et al., 2023), was not detected in this study. Its absence is likely due to its low relative abundance in grapevines. Previous research has shown that *Trichoderma* is a rare taxon in the grapevine wood microbiome (Del Frari et al., 2019). In line with our findings, other studies using DNA metabarcoding have also not detected *Trichoderma* in grapevine wood samples (Niem et al., 2020; Bekris et al., 2021; Liu and Howell, 2021).

Other grapevine microbiome investigations have reported some of the bacterial genera found in this study from grapevine trunks. For instance, Niem et al. (2020) identified bacteria such as *Pseudomonas, Sphingomonas, Hymenobacter*, and *Friedmanniella* when investigating the trunk endosphere of grapevines in Australian vineyards. In contrast to the findings of Niem et al. (2020), *Methylobacterium_Methylorubrum* and *Massilia* were reported from the grapevine trunk in this study, while *Hymenobacter* and *Friedmanniella* were reported in higher relative abundance. Many dominant bacteria reported in the Australian research, such as *Pedomicrobium*, *Xylanimicrobium*, and *Roseococcus*, were absent in this study. Similarly, Bruez et al. (2020) reported distinct bacterial taxa such as *Agrobacterium*, *Steroidobacter*, and *Janthinobacterium* in grapevine wood from France, which were not detected in this study. Nevertheless, the authors found similar bacteria, such as *Sphingomonas*, *Pseudomonas*, and *Pantoea*, in high relative abundance. *Bacillus* and *Streptomyces*, unique bacteria reported by Bekris et al. (2021) from grapevines in Greek vineyards, were not detected in this study. Distinct bacteria identified in this study might be attributed to New Zealand’s geographical isolation. This is consistent with the distance decay principle, which suggests that as the geographical distance between two plant locations increases, the microbial communities of those plants grow increasingly dissimilar (Hanson et al., 2012). Several studies have reported the impact of this distance decay on bacterial community composition (Mosqueira et al., 2019; Pu et al., 2022; Sun et al., 2022). The frequent isolation of bacteria such as *Pseudomonas, Pantoea, Curtobacterium*, and *Erwinia* in this study is supported by studies investigating the culturable bacterial community of grapevines (Bruez et al., 2015; Rezgui et al., 2016; Andreolli et al., 2023).

The microbiome structure differences between GTD escape and diseased vines were driven by *Pseudomonas* and *Hymenobacter*, which were relatively more abundant in the GTD escape vines. Three statistical tools also showed that *Pseudomonas* was discriminatory for GTD escape, while relative abundance data, edgeR, and Random Forest model, but not DESeq2, showed that *Hymenobacter* and *Massilia* were discriminatory for GTD escape. *Pseudomonas* is a diverse genus containing over 200 species, with many of them known to interact with plants (Guzmán-Guzmán and Santoyo, 2022). Multiple studies have used beneficial *Pseudomonas* species for the control of GTD pathogens (Rezgui et al., 2016; Wicaksono et al., 2017; Mesguida et al., 2023; Niem et al., 2023) and other plant pathogens (De Vrieze et al., 2018; Wicaksono et al., 2018; Minchev et al., 2021). In grapevines, some members of this genus have been associated with asymptomatic plants (Deyett et al., 2017; Niem et al., 2020).

Niem et al. (2020) demonstrated that *Pseudomonas* was the dominant bacterial genus in asymptomatic grapevines (up to 74% relative abundance) compared with grapevines showing GTD symptoms (up to 29% relative abundance). They further demonstrated that some *Pseudomonas* strains showed biological activity against GTD pathogens. Similarly, *Pseudomonas* was identified as the driver of disease escape from Pierce’s disease, where there was a negative correlation between *P. fluorescens* and the presence of the pathogen, *Xylella fastidiosa* (Deyett et al., 2017). *Pseudomonas fluorescens* isolates were subsequently shown to reduce disease development in *in planta* assays (Rolshausen et al., 2018). These beneficial *Pseudomonas* have several modes of action against pathogens, including direct antagonism (Ziedan and El-Mohamedy, 2008), production of siderophore, and induction of the plant immune response (Verhagen et al., 2010; Lakkis et al., 2019). The evidence from the current study and previous research support *Pseudomonas* as a genuine candidate driving the GTD escape phenotype.

We looked at the *Pseudomonas* taxon in more resolution because it was not consistently higher in relative abundance in GTD escape vines across the four vineyards. The analysis showed intriguing patterns across vineyards and indicated variations in the potential drivers of GTD escape within and across vineyards. For instance, while ASV 9 showed potential as a likely driver of GTD escape in vineyard 1, its inconsistent distribution in vineyards 2 and 3 complicates this interpretation. Similarly, ASV 71 was highly abundant in GTD escape samples in vineyards 3 and 4. However, the inconsistency in the trend in vineyards 1 and 2 further makes it challenging to conclude that the ASV is a GTD escape driver. In GTD escape vines, ASV 21 was detected at a higher relative abundance than diseased vines. This trend was observed across all four vineyards, with vineyards 1 and 2 showing particularly high relative abundance of the ASV in GTD escape vines. However, the ASV had low relative abundance in two vineyards, making the interpretation less straightforward. Despite this, its ubiquity and consistently higher relative abundance in GTD escape vines suggest it could be a key *Pseudomonas* species driving the GTD escape phenotype in the vineyards. The varied relative abundance patterns of these *Pseudomonas* ASVs showed that while unique *Pseudomonas* species may play significant roles in driving GTD escape in some vineyards, their influence may not be universal. This suggests that the roles of these individual *Pseudomonas* may be influenced by factors unique to each vineyard. Although we have identified the *Pseudomonas* genus as a driver of GTD escape, the specific lineage within the genus which acts as the primary driver of the GTD escape remains unclear. Some *Pseudomonas* likely possess unique genetic characteristics that enable them to drive GTD escape. We aim to understand this phenomenon better with future whole genome sequencing studies. The microbial isolations confirmed that we were able to isolate *Pseudomonas* similar to those detected in the DNA metabarcoding. For example, some *Pseudomonas* isolates clustered within groups that contained ASVs 8, 9, and 71, three of the ASVs with higher relative abundance in GTD escape vines. While our study identified an association between the *Pseudomonas* genus and the GTD escape phenotype across all samples, analysis of individual ASV showed that this phenotype may be driven by only few *Pseudomonas* species or subspecies. This varied diversity of *Pseudomonas* in individual vineyards highlights the importance of considering taxa with the ASV approach.

The bacterial taxon, *Hymenobacter*, was also significantly associated with GTD escape vines. There are few reports of *Hymenobacter*’s interactions with grapevines. Niem et al. (2020) found *Hymenobacter* in the endosphere of asymptomatic grapevine trunks but at a low relative abundance (1%). Although a previous study detected *Hymenobacter* sequences on grapevine foliage and grape berries (Aleynova et al., 2022), to our knowledge, the current study is the first time the taxon has been shown to be differentially associated with healthy grapevine trunks. In the Huanglongbing disease of citrus, the relative abundance of *Hymenobacter* was higher in the leaves of asymptomatic citrus (Blaustein et al., 2017). In citrus trees affected by low-level Huanglongbing disease, there was a higher relative abundance of *Hymenobacter* when the disease was in its early stages but a decrease as the disease progressed (Ginnan et al., 2020). In the current study, no *Hymenobacter* was isolated. Anguita-Maeso et al. (2020) also reported similar findings in their investigation of the olive xylem microbiota, where they found *Hymenobacter* with DNA metabarcoding yet could not isolate members of this genus from the plant samples. In the future, the medium and growth conditions could be optimized to enhance the isolation of *Hymenobacter* from grapevines. Further research is needed to understand how *Hymenobacter* interacts with grapevines, its associated microbiota, and the effects of the bacteria on the grapevine’s health.

Based on relative abundance data and statistical analysis, *Aureobasidium* and *Rhodotorula* emerged as two likely fungal drivers of the GTD escape phenotype. Specifically, *Aureobasidium* was indicated by the Random Forest analysis as the top taxon driving the GTD escape community. *Aureobasidium* is a yeast-like fungus that has been widely reported to be one of the most abundant fungal genera in grapevine leaves (Pinto et al., 2014), sap (Deyett and Rolshausen, 2019), trunk (Del Frari et al., 2019), flower, grapes, and roots (Liu and Howell, 2021). *Aureobasidium pullulans* has been reported to be significantly more abundant in the cordons of healthy grapevines than in symptomatic ones (Bruez et al., 2020). *Aureobasidium pullulans* has bioactive functions and has been tested against GTD pathogens in *in vitro* and *in planta* studies with promising results (Pinto et al., 2018; Blundell et al., 2021). The primary modes of action of *A. pullulans* are thought to be a direct antagonism against GTD pathogens and the stimulation of the grapevine’s immune response (Pinto et al., 2018). It also produces volatile organic compounds and hydrolytic enzymes, modulates pH unsuitable for pathogen growth, and competes against pathogens for nutrients and space (Di Francesco, 2015). Like *Aureobasidium*, *Rhodotorula* is a frequently occurring yeast found in grapevine leaves, berries and wood (Sipiczki, 2016; Del Frari et al., 2019; Liu and Howell, 2021; Costantini et al., 2022). *Rhodotorula rubra* has been shown to possess biological activity against *E. lata* (Munkvold and Marois, 1993) and the ability to produce auxins in plants (Kandel et al., 2017). These findings demonstrate the potential of members of these two genera in grapevine protection and growth promotion. It is important to note that the high relative abundance of *Eutypa* in many samples would have impacted the relative abundance of other taxa, possibly weakening the strength of our conclusions regarding these fungal taxa as drivers of the GTD escape phenotype.

*Phaeomoniella* was predominant in the Hawke’s Bay vineyards. Similar results were obtained in the studies of Niem et al. (2020) and Bruez et al. (2020), where *Phaeomoniella* had the highest relative abundance in Australian and French vineyards, respectively. Notably, *Phaeomoniella* showed a higher relative abundance in GTD escape than diseased vines across the vineyards. Despite its association with GTDs (Gramaje et al., 2018), *Phaeomoniella* is frequently found in symptomatic and asymptomatic grapevines. For example, Niem et al. (2020) reported a relatively higher abundance of *Phaeomoniella* in asymptomatic grapevine wood samples than in symptomatic samples. Similarly, Bruez et al. (2020) found more *Phaeomoniella* in healthy grapevine cordons than in diseased cordons. The trend in the relative abundance of *Phaeomoniella* in GTD escape and diseased vines could be attributed to its latent lifestyle. It has been hypothesized that esca-associated fungi, such as *Phaeomoniella*, were more likely to be endophytes or latent or nonspecific saprobes than pathogens (Hofstetter et al., 2012). Moreover, *Phaeomoniella* is mainly associated with Petri disease, which affects young grapevines and not mature grapevines, as sampled in this study, and esca disease, which has not been reported in New Zealand (Mundy and Manning, 2010; Urbez-Torres et al., 2014). *Phaeomoniella* was one of the abundant taxa in the metabarcoding data that was not isolated. Members of this genus can sometimes be difficult to isolate because of their slow-growing nature and the likelihood of fast-growing fungi overgrowing them (Retief et al., 2005; Bruez et al., 2014). Overall, there was no evidence of the involvement of *Phaeomoniella* in the GTDs observed in this study. In contrast, *Eutypa* was strongly involved in the GTDs observed.

The network analysis revealed compelling patterns in the microbial communities. The edges, which represent interactions within the network, were increased in the GTD escape networks. This, together with the consistently higher mean degree in the GTD escape networks, show that there were more interactions among the ASVs in GTD escape than diseased vines (Guo et al., 2022). Higher-degree nodes in the GTD escape networks may be essential for their community structure and function, as these nodes often represent keystone species (Berry and Widder, 2014). Among these high-degree nodes in the bacterial GTD escape network, we identified two taxa, *Mucilaginibacter* and *Massilia*, which were also present in the diseased network, albeit with different ASVs. The presence of both taxa in GTD escape and diseased networks and *Massilia’s* statistical association with GTD escape vines suggests that these taxa may play key roles in the grapevine microbiome. The differences in the ASVs of the taxa could mean unique lineages of the genera become keystone taxa under specific conditions, such as in the presence of GTDs. The higher relative modularity of the GTD escape networks suggests that the GTD escape vines may be more resilient to external disturbances like GTDs than the diseased vines (Hernandez et al., 2021). The concept of modularity in network analysis is the degree to which a network can be divided into distinct groups or modules. A modular network is considered more resilient because the external disturbances affecting a module are less likely to go throughout the network, therefore maintaining the network’s stability (Stouffer and Bascompte, 2011). These findings are further corroborated by the lower path length and diameter in the GTD escape networks, which indicates that there were closer and stronger relationships within the GTD escape network (Chen et al., 2021; Guo et al., 2022). The network analyses showed that GTDs negatively impact the complexity and stability of microbial networks in grapevines.

Despite considerable research efforts, there are still substantial knowledge gaps on the factors that contribute to GTDs, and the complex interactions among the grapevine host and its microbiota (Bertsch et al., 2013; Claverie et al., 2020). Consistent with the expectation that a GTD escape mechanism would not be uniform across vineyards, the taxonomic signals for GTD escape were observed only in three of the four vineyards. Future studies of GTD escape would be enhanced by expanding the scope of sampling from healthy vines under little or no disease pressure in vineyards so that comparisons can be made among GTD escape, healthy, and diseased vines. Finally, a primary limitation of DNA metabarcoding is the reliance on the relative abundance of taxa, which can be influenced by variations in the abundance of other taxa. Despite these limitations, key taxa associated with GTD escape were identified and distinct differences in the taxonomic composition of GTD escape and diseased vines on an individual vineyard level were observed. Identifying these trends, despite the variability in sample attributes such as vineyard locations and cultivars, suggests that the distinctions observed between GTD escape and diseased vines were genuine. Therefore, despite the potential influence of confounding factors, our study provides evidence linking the grapevine microbiome to the expression of the GTD escape phenotype in vineyards.

This study aimed to identify the microbiome structure of grapevines that escape GTDs and to uncover potential microbial contributions to the GTD escape phenotype. Prior to this investigation, reports of GTD escape were primarily based on anecdotal evidence. However, we show here that the fungal and bacterial microbiomes of GTD escape vines significantly differed from those of the diseased vines. Our analysis of the grapevine trunk microbiome revealed a significant abundance of microbial taxa in the GTD escape vines, such as *Pseudomonas, Hymenobacter, Massilia, Aureobasidium, Rhodotorula, Seimatosporium*, and *Cladosporium*. These results support previous studies that have linked plant microbiomes to the disease escape phenotype (Deyett et al., 2017; Kusstatscher et al., 2019; Li et al., 2022) and suggest that the microbial community of grapevines plays a critical role in the expression of the GTD escape phenotype. This functional linkage warrants further investigation to fully understand the mechanisms underlying the microbiome differences between GTD escape and diseased vines. The results of this study significantly advance our existing understanding of disease escape in plants, particularly highlighting the microbial communities associated with GTD escape vines and their potential roles in the prevention of GTDs.

## Data availability statement

The datasets presented in this study will be accessible at the following link after the indicated release date or on publication: https://www.ncbi.nlm.nih.gov/bioproject; PRJNA978637.

## Author contributions

DA drafted the manuscript, identified GTD escape vines, collected and processed plant samples, performed DNA extraction and metabarcoding, and analyzed and interpreted microbiome data. EJ and HR co-designed the study, assisted with data interpretation, and provided overall guidance for the project. DM offered expert advice on GTD and contributed to the identification of GTD escape vines and the collection of plant samples. BV contributed to the identification of GTD escape vines, DNA metabarcoding, and microbiome data analysis. SB co-designed the study, assisted in identifying GTD escape vines, helped to interpret data, and provided overall project guidance. All authors actively participated in reviewing and editing the manuscript and approved its final version.

## Funding

This work was funded by The New Zealand Institute of Plant and Food Research Ltd. (Strategic Science Investment Fund, Project P/471778/29). The funder was not involved in the study design, collection, analysis, interpretation of data, the writing of this article or the decision to submit it for publication. DA received a writing scholarship from the Faculty of Agriculture and Life Science, Lincoln University. This publication has been partially financed by the Lincoln University Open Access Fund.

## Conflict of interest

HR, DM, and SB are employed by The New Zealand Institute for Plant and Food Research Limited. BV was employed by The New Zealand Institute for Plant and Food Research Limited and is currently employed by Bragato Research Institute.

The remaining authors declare that the research was conducted in the absence of any commercial or financial relationships that could be construed as a potential conflict of interest.

## Publisher’s note

All claims expressed in this article are solely those of the authors and do not necessarily represent those of their affiliated organizations, or those of the publisher, the editors and the reviewers. Any product that may be evaluated in this article, or claim that may be made by its manufacturer, is not guaranteed or endorsed by the publisher.

## Footnotes

^1^ http://cliflo.niwa.co.nz

## References

[ref1] Aleynova O. A. Nityagovsky N. N. Dubrovina A. S. Kiselev K. V. (2022). The biodiversity of grapevine bacterial endophytes of *Vitis amurensis* rupr. Plan. Theory 11:1128. doi: 10.3390/plants11091128 PMC9099740 35567129

[ref2] Altschul S. F. Madden T. L. Schäffer A. A. Zhang J. Zhang Z. Miller W.. (1997). Gapped BLAST and PSI-BLAST: a new generation of protein database search programs. Nucleic Acids Res. 25, 3389–3402. 9254694 10.1093/nar/25.17.3389 PMC146917

[ref3] Andreolli M. Lampis S. Tosi L. Marano V. Zapparoli G. (2023). Fungicide sensitivity of grapevine bacteria with plant growth-promoting traits and antagonistic activity as non-target microorganisms. World J. Microbiol. Biotechnol. 39:121. doi: 10.1007/s11274-023-03569-5 36929028 PMC10020324

[ref4] Anguita-Maeso M. Olivares-García C. Haro C. Imperial J. Navas-Cortés J. A. Landa B. B. (2020). Culture-dependent and culture-independent characterization of the olive xylem microbiota: effect of sap extraction methods. Front. Plant Sci. 10:1708. doi: 10.3389/fpls.2019.01708 32038682 PMC6988092

[ref5] Araújo W. L. Marcon J. Maccheroni W. van Elsas J. D. van Vuurde J. W. Azevedo J. L. (2002). Diversity of endophytic bacterial populations and their interaction with *Xylella fastidiosa* in citrus plants. Appl. Environ. Microbiol. 68, 4906–4914. doi: 10.1128/AEM.68.10.4906-4914.2002 12324338 PMC126398

[ref6] Bekris F. Vasileiadis S. Papadopoulou E. Samaras A. Testempasis S. Gkizi D.. (2021). Grapevine wood microbiome analysis identifies key fungal pathogens and potential interactions with the bacterial community implicated in grapevine trunk disease appearance. Environ. Microbiome 16, 1–17. doi: 10.1186/s40793-022-00405-5 34863281 PMC8642934

[ref7] Berendsen R. L. Pieterse C. M. Bakker P. A. (2012). The rhizosphere microbiome and plant health. Trends Plant Sci. 17, 478–486. doi: 10.1016/j.tplants.2012.04.001 22564542

[ref8] Berry D. Widder S. (2014). Deciphering microbial interactions and detecting keystone species with co-occurrence networks. Front. Microbiol. 5:219. doi: 10.3389/fmicb.2014.00219 24904535 PMC4033041

[ref9] Bertsch C. Ramírez-Suero M. Magnin-Robert M. Larignon P. Chong J. Abou-Mansour E.. (2013). Grapevine trunk diseases: complex and still poorly understood. Plant Pathol. 62, 243–265. doi: 10.1111/j.1365-3059.2012.02674.x

[ref10] Bettenfeld P. Fontaine F. Trouvelot S. Fernandez O. Courty P.-E. (2020). Woody plant declines. What's wrong with the microbiome? Trends Plant Sci. 25, 381–394. doi: 10.1016/j.tplants.2019.12.024 31983620

[ref11] Bettenfeld P. ICanals J. C. Jacquens L. Fernandez O. Fontaine F. van Schaik E.. (2022). The microbiota of the grapevine holobiont: a key component of plant health. J. Adv. Res. 40, 1–15. doi: 10.1016/j.jare.2021.12.008 36100319 PMC9481934

[ref12] Beule L. Karlovsky P. (2020). Improved normalization of species count data in ecology by scaling with ranked subsampling (SRS): application to microbial communities. PeerJ 8:e9593. doi: 10.7717/peerj.9593 32832266 PMC7409812

[ref13] Blaustein R. A. Lorca G. L. Meyer J. L. Gonzalez C. F. Teplitski M. (2017). Defining the core citrus leaf- and root-associated microbiota: factors associated with community structure and implications for managing Huanglongbing (citrus greening) disease. Appl. Environ. Microbiol. 83:e00210-17. doi: 10.1128/AEM.00210-17 28341678 PMC5440699

[ref14] Blundell R. Arreguin M. Eskalen A. (2021). *In vitro* evaluation of grapevine endophytes, epiphytes and sap micro-organisms for potential use to control grapevine trunk disease pathogens. Phytopathol. Mediterr. 60, 535–548. doi: 10.36253/phyto-12500

[ref15] Bodenhausen N. Horton M. W. Bergelson J. (2013). Bacterial communities associated with the leaves and the roots of *Arabidopsis thaliana*. PLoS One 8:e56329. doi: 10.1371/journal.pone.0056329 23457551 PMC3574144

[ref16] Bonito G. Hameed K. Ventura R. Krishnan J. Schadt C. W. Vilgalys R. (2016). Isolating a functionally relevant guild of fungi from the root microbiome of *Populus*. Fungal Ecol. 22, 35–42. doi: 10.1016/j.funeco.2016.04.007

[ref17] Breiman L. (2001). Random forests. Mach. Learn. 45, 5–32. doi: 10.1023/A:1010933404324

[ref18] Bruez E. Baumgartner K. Bastien S. Travadon R. Guérin-Dubrana L. Rey P. (2016). Various fungal communities colonise the functional wood tissues of old grapevines externally free from grapevine trunk disease symptoms. Aust. J. Grape Wine Res. 22, 288–295. doi: 10.1111/ajgw.12209

[ref19] Bruez E. Haidar R. Alou M. T. Vallance J. Bertsch C. Mazet F.. (2015). Bacteria in a wood fungal disease: characterization of bacterial communities in wood tissues of esca-foliar symptomatic and asymptomatic grapevines. Front. Microbiol. 6:1137. doi: 10.3389/fmicb.2015.01137 26579076 PMC4621878

[ref20] Bruez E. Vallance J. Gautier A. Laval V. Compant S. Maurer W.. (2020). Major changes in grapevine wood microbiota are associated with the onset of esca, a devastating trunk disease. Environ. Microbiol. 22, 5189–5206. doi: 10.1111/1462-2920.15180 32755016

[ref21] Bruez E. Vallance J. Gerbore J. Lecomte P. Da Costa J.-P. Guerin-Dubrana L.. (2014). Analyses of the temporal dynamics of fungal communities colonizing the healthy wood tissues of esca leaf-symptomatic and asymptomatic vines. PLoS One 9:e95928. doi: 10.1371/journal.pone.0095928 24788412 PMC4006835

[ref22] Callahan B. J. McMurdie P. J. Rosen M. J. Han A. W. Johnson A. J. A. Holmes S. P. (2016). DADA2: high-resolution sample inference from Illumina amplicon data. Nat. Methods 13, 581–583. doi: 10.1038/nmeth.3869 27214047 PMC4927377

[ref23] Calvo-Garrido C. Songy A. Marmol A. Roda R. Clément C. Fontaine F. (2021). Description of the relationship between trunk disease expression and meteorological conditions, irrigation and physiological response in chardonnay grapevines. OENO One 55, 97–113. doi: 10.20870/oeno-one.2021.55.2.4548

[ref24] Carter M. V. (1991). The status of *Eutypa lata* as a pathogen. Phytopathology Paper 32:59.

[ref25] Chappell P.R. (2013). The climate and weather of Hawke’s bay. NIWA Science and Technology Series. 3rd Ed.

[ref26] Chelius M. Triplett E. (2001). The diversity of Archaea and bacteria in association with the roots of *Zea mays* L. Microb. Ecol. 41, 252–263. doi: 10.1007/s002480000087 11391463

[ref27] Chen B. Jiao S. Luo S. Ma B. Qi W. Cao C.. (2021). High soil pH enhances the network interactions among bacterial and archaeal microbiota in alpine grasslands of the Tibetan Plateau. Environ. Microbiol. 23, 464–477. doi: 10.1111/1462-2920.15333 33215802

[ref28] Chong J. Liu P. Zhou G. Xia J. (2020). Using MicrobiomeAnalyst for comprehensive statistical, functional, and meta-analysis of microbiome data. Nat. Protoc. 15, 799–821. doi: 10.1038/s41596-019-0264-1 31942082

[ref29] Claverie M. Notaro M. Fontaine F. Wery J. (2020). Current knowledge on grapevine trunk diseases with complex etiology: a systemic approach. Phytopathol. Mediterr. 59, 29–53. doi: 10.36253/phyto-11150

[ref30] Compant S. Samad A. Faist H. Sessitsch A. (2019). A review on the plant microbiome: ecology, functions and emerging trends in microbial application. J. Adv. Res. 19, 29–37. doi: 10.1016/j.jare.2019.03.004 31341667 PMC6630030

[ref31] Costantini A. Vaudano E. Pulcini L. Boatti L. Gamalero E. Garcia-Moruno E. (2022). Yeast biodiversity in vineyard during grape ripening: comparison between culture dependent and NGS analysis. PRO 10:901. doi: 10.3390/pr10050901

[ref32] De La Fuente M. Fontaine F. Gramaje D. Armengol J. Smart R. Nagy Z.A.. (2016). Grapevine trunk diseases. A review. 1st. (Paris, France: International Organisation of Vine and Wine (OIV)).

[ref33] De Vrieze M. Germanier F. Vuille N. Weisskopf L. (2018). Combining different potato-associated *Pseudomonas* strains for improved biocontrol of *Phytophthora infestans*. Front. Microbiol. 9:2573. doi: 10.3389/fmicb.2018.02573 30420845 PMC6215842

[ref34] Del Frari G. Gobbi A. Aggerbeck M. R. Oliveira H. Hansen L. H. Ferreira R. B. (2019). Characterization of the wood mycobiome of *Vitis vinifera* in a vineyard affected by esca. Spatial distribution of fungal communities and their putative relation with leaf symptoms. Front. Plant Sci. 10:910. doi: 10.3389/fpls.2019.00910 31354777 PMC6640213

[ref35] Deyett E. Rolshausen P. E. (2019). Temporal dynamics of the sap microbiome of grapevine under high Pierce's disease pressure. Front. Plant Sci. 10:1246. doi: 10.3389/fpls.2019.01246 31681363 PMC6805966

[ref36] Deyett E. Roper M. C. Ruegger P. Yang J.-I. Borneman J. Rolshausen P. E. (2017). Microbial landscape of the grapevine endosphere in the context of Pierce's disease. Phytobiomes J. 1, 138–149. doi: 10.1094/pbiomes-08-17-0033-r

[ref37] Di Francesco A. (2015). Aureobasidium pullulans as biological control agent: modes of action. PhD. thesis. Bologna: University of Bologna.

[ref38] Edgar R. C. (2004). MUSCLE: multiple sequence alignment with high accuracy and high throughput. Nucleic Acids Res. 32, 1792–1797. doi: 10.1093/nar/gkh340 15034147 PMC390337

[ref39] Egamberdieva D. Wirth S. J. Alqarawi A. A. Abd_Allah E. F. Hashem A. (2017). Phytohormones and beneficial microbes: essential components for plants to balance stress and fitness. Front. Microbiol. 8:2104. doi: 10.3389/fmicb.2017.02104 29163398 PMC5671593

[ref40] Friedman J. Alm E. J. (2012). Inferring correlation networks from genomic survey data. PLoS Comput. Biol. 8:e1002687. doi: 10.1371/journal.pcbi.1002687 23028285 PMC3447976

[ref41] Gdanetz K. Trail F. (2017). The wheat microbiome under four management strategies, and potential for endophytes in disease protection. Phytobiomes J. 1, 158–168. doi: 10.1094/pbiomes-05-17-0023-r

[ref42] Ginnan N. A. Dang T. Bodaghi S. Ruegger P. M. McCollum G. England G.. (2020). Disease-induced microbial shifts in citrus indicate microbiome-derived responses to Huanglongbing across the disease severity spectrum. Phytobiomes J. 4, 375–387. doi: 10.1094/pbiomes-04-20-0027-r

[ref43] González V. Tello M. L. (2011). The endophytic mycota associated with *Vitis vinifera* in Central Spain. Fungal Divers. 47, 29–42. doi: 10.1007/s13225-010-0073-x

[ref44] Gramaje D. Urbez-Torres J. R. Sosnowski M. R. (2018). Managing grapevine trunk diseases with respect to etiology and epidemiology: current strategies and future prospects. Plant Dis. 102, 12–39. doi: 10.1094/PDIS-04-17-0512-FE 30673457

[ref46] Guo B. Zhang L. Sun H. Gao M. Yu N. Zhang Q.. (2022). Microbial co-occurrence network topological properties link with reactor parameters and reveal importance of low-abundance genera. NPJ Biofilms Microbiomes 8:3. doi: 10.1038/s41522-021-00263-y 35039527 PMC8764041

[ref47] Guzmán-Guzmán P. Santoyo G. (2022). Action mechanisms, biodiversity, and omics approaches in biocontrol and plant growth-promoting Pseudomonas: an updated review. Biocontrol Sci. Tech. 32, 527–550. doi: 10.1080/09583157.2022.2066630

[ref48] Hanson C. A. Fuhrman J. A. Horner-Devine M. C. Martiny J. B. H. (2012). Beyond biogeographic patterns: processes shaping the microbial landscape. Nat. Rev. Microbiol. 10, 497–506. doi: 10.1038/nrmicro2795 22580365

[ref49] Hernandez D. J. David A. S. Menges E. S. Searcy C. A. Afkhami M. E. (2021). Environmental stress destabilizes microbial networks. ISME J. 15, 1722–1734. doi: 10.1038/s41396-020-00882-x 33452480 PMC8163744

[ref50] Hofstetter V. Buyck B. Croll D. Viret O. Couloux A. Gindro K. (2012). What if esca disease of grapevine were not a fungal disease? Fungal Divers. 54, 51–67. doi: 10.1007/s13225-012-0171-z

[ref51] Hrycan J. Hart M. Bowen P. Forge T. Urbez-Torres J. R. (2020). Grapevine trunk disease fungi: their roles as latent pathogens and stress factors that favour disease development and symptom expression. Phytopathol. Mediterr. 59, 395–424.

[ref52] Kandel S. L. Firrincieli A. Joubert P. M. Okubara P. A. Leston N. D. McGeorge K. M.. (2017). An in vitro study of bio-control and plant growth promotion potential of Salicaceae endophytes. Front. Microbiol. 8:386. doi: 10.3389/fmicb.2017.00386 28348550 PMC5347143

[ref53] Khan M. I. Marroni V. Keenan S. Scott I. A. W. Viljanen-Rollinson S. L. H. Bulman S. (2013). Enhanced molecular identification of Botrytis spp. from New Zealand onions. Eur. J. Plant Pathol. 136, 495–507. doi: 10.1007/s10658-013-0182-y

[ref54] Kraus C. Voegele R. T. Fischer M. (2019). Temporal development of the culturable, endophytic fungal community in healthy grapevine branches and occurrence of GTD-associated fungi. Microb. Ecol. 77, 866–876. doi: 10.1007/s00248-018-1280-3 30397796

[ref55] Kusstatscher P. Cernava T. Harms K. Maier J. Eigner H. Berg G.. (2019). Disease incidence in sugar beet fields is correlated with microbial diversity and distinct biological markers. Phytobiomes J. 3, 22–30. doi: 10.1094/pbiomes-01-19-0008-r

[ref56] Lakkis S. Trotel-Aziz P. Rabenoelina F. Schwarzenberg A. Nguema-Ona E. Clément C.. (2019). Strengthening grapevine resistance by *Pseudomonas fluorescens* pta-ct2 relies on distinct defense pathways in susceptible and partially resistant genotypes to downy mildew and gray mold diseases. Front. Plant Sci. 10:1112. doi: 10.3389/fpls.2019.01112 31620150 PMC6759587

[ref57] Li Y. He F. Guo Q. Feng Z. Zhang M. Ji C.. (2022). Compositional and functional comparison on the rhizosphere microbial community between healthy and Sclerotium rolfsii-infected monkshood (*Aconitum carmichaelii*) revealed the biocontrol potential of healthy monkshood rhizosphere microorganisms. Biol. Control 165:104790. doi: 10.1016/j.biocontrol.2021.104790

[ref58] Liaw A. Wiener M. (2002). Classification and regression by randomForest. R News 2, 18–22.

[ref59] Liu D. Howell K. (2021). Community succession of the grapevine fungal microbiome in the annual growth cycle. Environ. Microbiol. 23, 1842–1857. doi: 10.1111/1462-2920.15172 32686214

[ref60] Lòpez-Fernàndez S. Mazzoni V. Pedrazzoli F. Pertot I. Campisano A. (2017). A phloem-feeding insect transfers bacterial endophytic communities between grapevine plants. Front. Microbiol. 8:834. doi: 10.3389/fmicb.2017.00834 28555131 PMC5430944

[ref61] Love M. Anders S. Huber W. (2014). Differential analysis of count data–the DESeq2 package. Genome Biol. 15, 10–1186. doi: 10.1101/002832

[ref62] Macara G.R. (2016). The climate and weather of Canterbury. NIWA Science and Technology Series. 2nd Ed.

[ref63] McKnight D. T. Huerlimann R. Bower D. S. Schwarzkopf L. Alford R. A. Zenger K. R. (2019). microDecon: a highly accurate read-subtraction tool for the post-sequencing removal of contamination in metabarcoding studies. Environ. DNA 1, 14–25. doi: 10.1002/edn3.11

[ref64] McLaren M. R. Callahan B. J. (2020). Pathogen resistance may be the principal evolutionary advantage provided by the microbiome. Philos. Trans. R. Soc. B 375:20190592. doi: 10.1098/rstb.2019.0592 PMC7435163 32772671

[ref65] McMurdie P. J. Holmes S. (2013). phyloseq: an R package for reproducible interactive analysis and graphics of microbiome census data. PLoS One 8:e61217. doi: 10.1371/journal.pone.0061217 23630581 PMC3632530

[ref66] McMurdie P. J. Holmes S. (2014). Waste not, want not: why rarefying microbiome data is inadmissible. PLoS Comput. Biol. 10:e1003531. doi: 10.1371/journal.pcbi.1003531 24699258 PMC3974642

[ref67] Mesguida O. Haidar R. Yacoub A. Dreux-Zigha A. Berthon J.-Y. Guyoneaud R.. (2023). Microbial biological control of fungi associated with grapevine trunk diseases: a review of strain diversity, modes of action, and advantages and limits of current strategies. Journal of Fungi 9:638. doi: 10.3390/jof9060638 37367574 PMC10299619

[ref68] Minchev Z. Kostenko O. Soler R. Pozo M. J. (2021). Microbial consortia for effective biocontrol of root and foliar diseases in tomato. Front. Plant Sci. 12:756368. doi: 10.3389/fpls.2021.756368 34804094 PMC8602810

[ref69] Mondello V. Songy A. Battiston E. Pinto C. Coppin C. Trotel-Aziz P.. (2018). Grapevine trunk diseases: a review of fifteen years of trials for their control with chemicals and biocontrol agents. Plant Dis. 102, 1189–1217. doi: 10.1094/PDIS-08-17-1181-FE 30673583

[ref70] Mosqueira M. J. Marasco R. Fusi M. Michoud G. Merlino G. Cherif A.. (2019). Consistent bacterial selection by date palm root system across heterogeneous desert oasis agroecosystems. Sci. Rep. 9:4033. doi: 10.1038/s41598-019-40551-4 30858421 PMC6412053

[ref71] Mueller U. G. Sachs J. L. (2015). Engineering microbiomes to improve plant and animal health. Trends Microbiol. 23, 606–617. doi: 10.1016/j.tim.2015.07.009 26422463

[ref72] Mundy D. C. Brown A. Jacobo F. Tennakoon K. Woolley R. H. Vanga B.. (2020). Pathogenic fungi isolated in association with grapevine trunk diseases in New Zealand. N. Z. J. Crop. Hortic. Sci. 48, 84–96. doi: 10.1080/01140671.2020.1716813

[ref73] Mundy D. C. Manning M. A. (2010). Ecology and management of grapevine trunk diseases in New Zealand: a review. New Zealand Plant Protection 63, 160–166. doi: 10.30843/nzpp.2010.63.6558

[ref74] Mundy D. C. Vanga B. R. Thompson S. Bulman S. (2018). Assessment of sampling and DNA extraction methods for identification of grapevine trunk microorganisms using metabarcoding. New Zealand Plant Protection 71, 10–18. doi: 10.30843/nzpp.2018.71.159

[ref75] Munkvold G. Marois J. (1993). Efficacy of natural epiphytes and colonizers of grapevine pruning wounds for biological control of Eutypa dieback. Phytopathology 83, 624–629.

[ref76] Niem J. Baaijens R. Savocchia S. Stodart B. Reveglia P. (2023). Biocontrol potential of an endophytic *Pseudomonas poae* strain against the grapevine trunk disease pathogen Neofusicoccum luteum and its mechanism of action. Plant Theory 12:2132. doi: 10.3390/plants12112132 PMC10255278 37299111

[ref77] Niem J. M. Billones-Baaijens R. Stodart B. Savocchia S. (2020). Diversity profiling of grapevine microbial endosphere and antagonistic potential of endophytic Pseudomonas against grapevine trunk diseases. Front. Microbiol. 11, 1–19. doi: 10.3389/fmicb.2020.00477 32273871 PMC7113392

[ref78] Nilsson R. H. Larsson K.-H. Taylor A. F. S. Bengtsson-Palme J. Jeppesen T. S. Schigel D.. (2019). The UNITE database for molecular identification of fungi: handling dark taxa and parallel taxonomic classifications. Nucleic Acids Res. 47, 259–264. doi: 10.1093/nar/gky1022 PMC6324048 30371820

[ref79] Oksanen J. (2010). Vegan: community ecology package. Available at: http://CRAN.R-project.org/package=vegan.

[ref80] Olesen J. M. Bascompte J. Dupont Y. L. Jordano P. (2007). The modularity of pollination networks. Proc. Natl. Acad. Sci. 104, 19891–19896. doi: 10.1073/pnas.0706375104 18056808 PMC2148393

[ref81] Paolinelli M. Escoriaza G. Cesari C. Garcia-Lampasona S. Hernandez-Martinez R. (2022). Characterization of grapevine wood microbiome through a metatranscriptomic approach. Microb. Ecol. 83, 658–668. doi: 10.1007/s00248-021-01801-z 34191105

[ref82] Paulson J. N. Stine O. C. Bravo H. C. Pop M. (2013). Differential abundance analysis for microbial marker-gene surveys. Nat. Methods 10, 1200–1202. doi: 10.1038/nmeth.2658 24076764 PMC4010126

[ref83] Pinto C. Custódio V. Nunes M. Songy A. Rabenoelina F. Courteaux B.. (2018). Understand the potential role of Aureobasidium pullulans, a resident microorganism from grapevine, to prevent the infection caused by Diplodia seriata. Front. Microbiol. 9:3047. doi: 10.3389/fmicb.2018.03047 30619138 PMC6297368

[ref84] Pinto C. Pinho D. Sousa S. Pinheiro M. Egas C. Gomes A. C. (2014). Unravelling the diversity of grapevine microbiome. PLoS One 9:e85622. doi: 10.1371/journal.pone.0085622 24454903 PMC3894198

[ref85] Pu T. Liu J. Dong J. Qian J. Zhou Z. Xia C.. (2022). Microbial community diversity and function analysis of *Aconitum carmichaelii* Debeaux in rhizosphere soil of farmlands in Southwest China. Front. Microbiol. 13:1055638. doi: 10.3389/fmicb.2022.1055638 36590406 PMC9797738

[ref86] Quast C. Pruesse E. Yilmaz P. Gerken J. Schweer T. Yarza P.. (2012). The SILVA ribosomal RNA gene database project: improved data processing and web-based tools. Nucleic Acids Res. 41, 590–596. doi: 10.1093/nar/gks1219 PMC3531112 23193283

[ref87] R Core Team (2013). R: A language and environment for statistical computing. R Foundation for Statistical Computing, Vienna, Austria.

[ref88] Retief E. Damm U. Van Niekerk J. McLeod A. Fourie P. (2005). A protocol for molecular detection of Phaeomoniella chlamydospora in grapevine wood: research in action. S. Afr. J. Sci. 101, 139–142.

[ref89] Rezgui A. Ben Ghnaya-Chakroun A. Vallance J. Bruez E. Hajlaoui M. R. Sadfi-Zouaoui N.. (2016). Endophytic bacteria with antagonistic traits inhabit the wood tissues of grapevines from Tunisian vineyards. Biol. Control 99, 28–37. doi: 10.1016/j.biocontrol.2016.04.005

[ref90] Riera N. Handique U. Zhang Y. Dewdney M. M. Wang N. (2017). Characterization of antimicrobial-producing beneficial bacteria isolated from Huanglongbing escape citrus trees. Front. Microbiol. 8:2415. doi: 10.3389/fmicb.2017.02415 29375487 PMC5770638

[ref91] Robinson M. D. McCarthy D. J. Smyth G. K. (2010). edgeR: a Bioconductor package for differential expression analysis of digital gene expression data. Bioinformatics 26, 139–140. doi: 10.1093/bioinformatics/btp616 19910308 PMC2796818

[ref92] Rolshausen P. Roper C. Maloney K. (2018). Greenhouse evaluation of grapevine microbial endophytes and fungal natural products for control of Pierce’s disease. Final report of CDFA Agreement Number 16‐0512‐SA. Available at: http://www.piercedisease.org.

[ref93] Saitou N. Nei M. (1987). The neighbor-joining method: a new method for reconstructing phylogenetic trees. Mol. Biol. Evol. 4, 406–425. 3447015 10.1093/oxfordjournals.molbev.a040454

[ref94] Sipiczki M. (2016). Overwintering of vineyard yeasts: survival of interacting yeast communities in grapes mummified on vines. Front. Microbiol. 7:212. doi: 10.3389/fmicb.2016.00212 26973603 PMC4770031

[ref95] Slowikowski K. (2018). ggrepel: automatically position non-overlapping text labels with "ggplot2.". R package version 0.8. 0 ed.

[ref96] Songy A. Fernandez O. Clement C. Larignon P. Fontaine F. (2019). Grapevine trunk diseases under thermal and water stresses. Planta 249, 1655–1679. doi: 10.1007/s00425-019-03111-8 30805725

[ref97] Sosnowski M. Shtienberg D. Creaser M. Wicks T. Lardner R. Scott E. (2007). The influence of climate on foliar symptoms of Eutypa dieback in grapevines. Phytopathology 97, 1284–1289. doi: 10.1094/PHYTO-97-10-1284 18943686

[ref98] Stouffer D. B. Bascompte J. (2011). Compartmentalization increases food-web persistence. Proc. Natl. Acad. Sci. 108, 3648–3652. doi: 10.1073/pnas.1014353108 21307311 PMC3048152

[ref99] Sun X. Zhang X. Zhang G. Miao Y. Zeng T. Zhang M.. (2022). Environmental response to root secondary metabolite accumulation in *Paeonia lactiflora*: insights from rhizosphere metabolism and root-associated microbial communities. Microbiol. Spectr. 10:e0280022. doi: 10.1128/spectrum.02800-22 36318022 PMC9769548

[ref100] Tamura K. Stecher G. Kumar S. (2021). MEGA11: molecular evolutionary genetics analysis version 11. Mol. Biol. Evol. 38, 3022–3027. doi: 10.1093/molbev/msab120 33892491 PMC8233496

[ref101] Travadon R. Lecomte P. Diarra B. Lawrence D. P. Renault D. Ojeda H.. (2016). Grapevine pruning systems and cultivars influence the diversity of wood-colonizing fungi. Fungal Ecol. 24, 82–93. doi: 10.1016/j.funeco.2016.09.003

[ref102] Trivedi P. Leach J. E. Tringe S. G. Sa T. Singh B. K. (2020). Plant–microbiome interactions: from community assembly to plant health. Nat. Rev. Microbiol. 18, 607–621. doi: 10.1038/s41579-020-0412-1 32788714

[ref103] Turner T. R. James E. K. Poole P. S. (2013). The plant microbiome. Genome Biol. 14:209. doi: 10.1186/gb-2013-14-6-209 23805896 PMC3706808

[ref104] Urbez-Torres J. R. Haag P. Bowen P. O'Gorman D. T. (2014). Grapevine trunk diseases in British Columbia: incidence and characterization of the fungal pathogens associated with black foot disease of grapevine. Plant Dis. 98, 456–468. doi: 10.1094/PDIS-05-13-0524-RE 30708694

[ref105] Vanga B. R. Panda P. Shah A. S. Thompson S. Woolley R. H. Ridgway H. J.. (2022). DNA metabarcoding reveals high relative abundance of trunk disease fungi in grapevines from Marlborough, New Zealand. BMC Microbiol. 22:126. doi: 10.1186/s12866-022-02520-2 35538413 PMC9088082

[ref106] Verhagen B. W. Trotel-Aziz P. Couderchet M. Höfte M. Aziz A. (2010). Pseudomonas spp.-induced systemic resistance to Botrytis cinerea is associated with induction and priming of defence responses in grapevine. J. Exp. Bot. 61, 249–260. doi: 10.1093/jxb/erp295 19812243

[ref107] Wang N. Stelinski L. L. Pelz-Stelinski K. S. Graham J. H. Zhang Y. (2017). Tale of the huanglongbing disease pyramid in the context of the citrus microbiome. Phytopathology 107, 380–387. doi: 10.1094/PHYTO-12-16-0426-RVW 28095208

[ref108] Weisburg W. G. Barns S. M. Pelletier D. A. Lane D. J. (1991). 16S ribosomal DNA amplification for phylogenetic study. J. Bacteriol. 173, 697–703. 1987160 10.1128/jb.173.2.697-703.1991 PMC207061

[ref109] Wen T. Xie P. Yang S. Niu G. Liu X. Ding Z.. (2022). ggClusterNet: an R package for microbiome network analysis and modularity-based multiple network layouts. iMeta 1:e32. doi: 10.1002/imt2.32 PMC10989811 38868720

[ref110] Wicaksono W. A. Jones E. E. Casonato S. Monk J. Ridgway H. J. (2018). Biological control of *Pseudomonas syringae* pv. actinidiae (Psa), the causal agent of bacterial canker of kiwifruit, using endophytic bacteria recovered from a medicinal plant. Biol. Control 116, 103–112. doi: 10.1016/j.biocontrol.2017.03.003

[ref111] Wicaksono W. A. Jones E. E. Monk J. Ridgway H. J. (2017). Using bacterial endophytes from a New Zealand native medicinal plant for control of grapevine trunk diseases. Biol. Control 114, 65–72. doi: 10.1016/j.biocontrol.2017.08.003

[ref112] Xia Y. Sun J. Chen D.-G. (2018). Statistical analysis of microbiome data with R. Singapore: Springer.

[ref113] Yuan M. M. Guo X. Wu L. Zhang Y. Xiao N. Ning D.. (2021). Climate warming enhances microbial network complexity and stability. Nat. Clim. Chang. 11, 343–348. doi: 10.1038/s41558-021-00989-9

[ref114] Zeng Q. Man X. Dai Y. Liu H. (2022). Pseudomonas spp. enriched in endophytic community of healthy cotton plants inhibit cotton Verticillium wilt. Front. Microbiol. 13:906732. doi: 10.3389/fmicb.2022.906732 35923406 PMC9339998

[ref115] Ziedan E. El-Mohamedy R. (2008). Application of *Pseudomonas fluorescens* for controlling root-rot disease of grapevine. Res. J. Agric. Biol. Sci. 4, 346–353.

