# Supplementary material for: Grapevines escaping trunk diseases in New Zealand vineyards have a distinct microbiome structure
Source: Front Microbiol. 2023 Aug 23;14:1231832. doi: 10.3389/fmicb.2023.1231832 (PMC10482235; doi:10.3389/fmicb.2023.1231832)
Supplement: Supplementary file 1 [file Data_Sheet_1.zip › Supplementary File 1.pptx]

## Slide 1
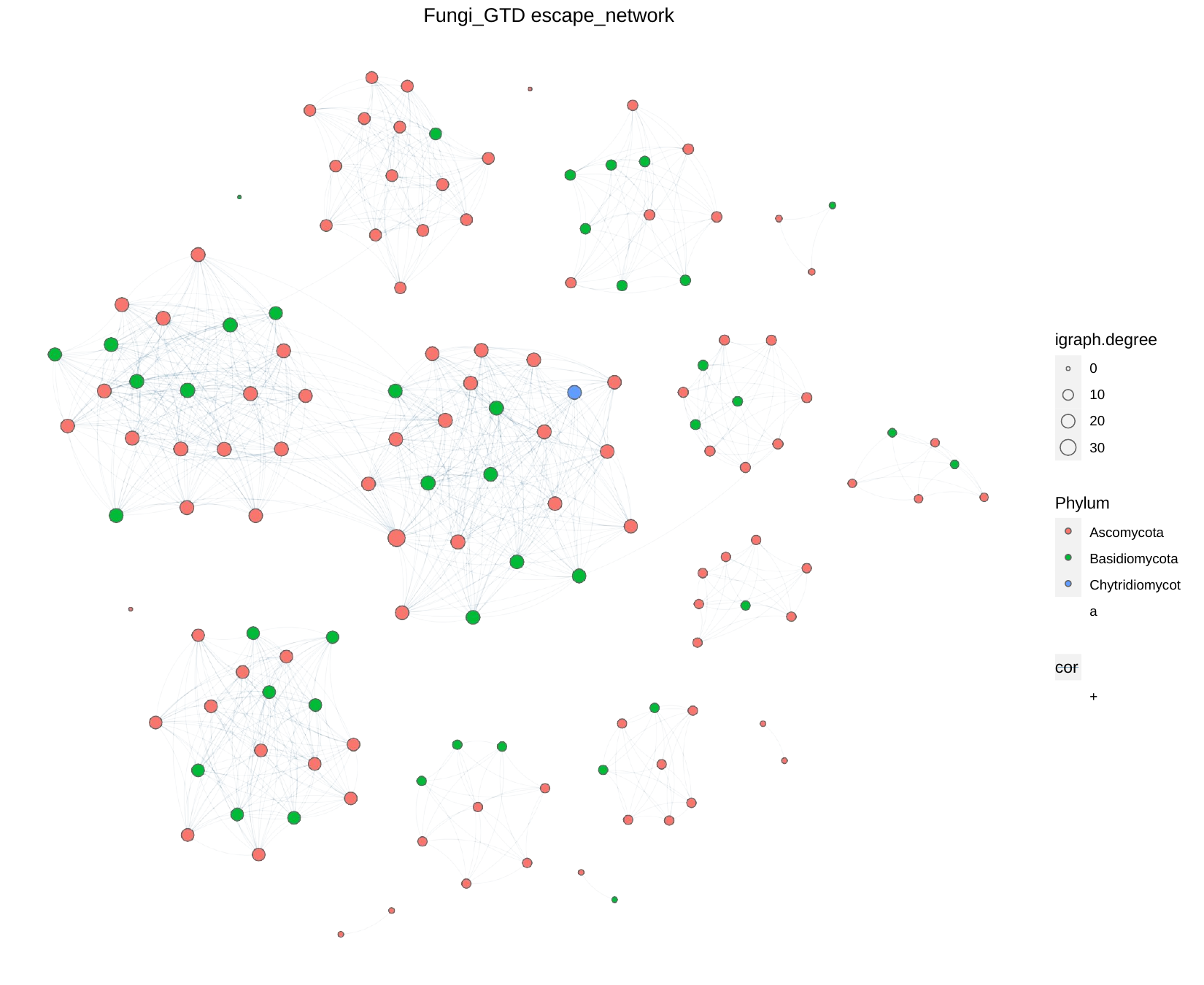

Fungi_GTD escape_network
igraph.degree
0
10
20
30
Phylum
Ascomycota Basidiomycota Chytridiomycota
cor
+

## Slide 2
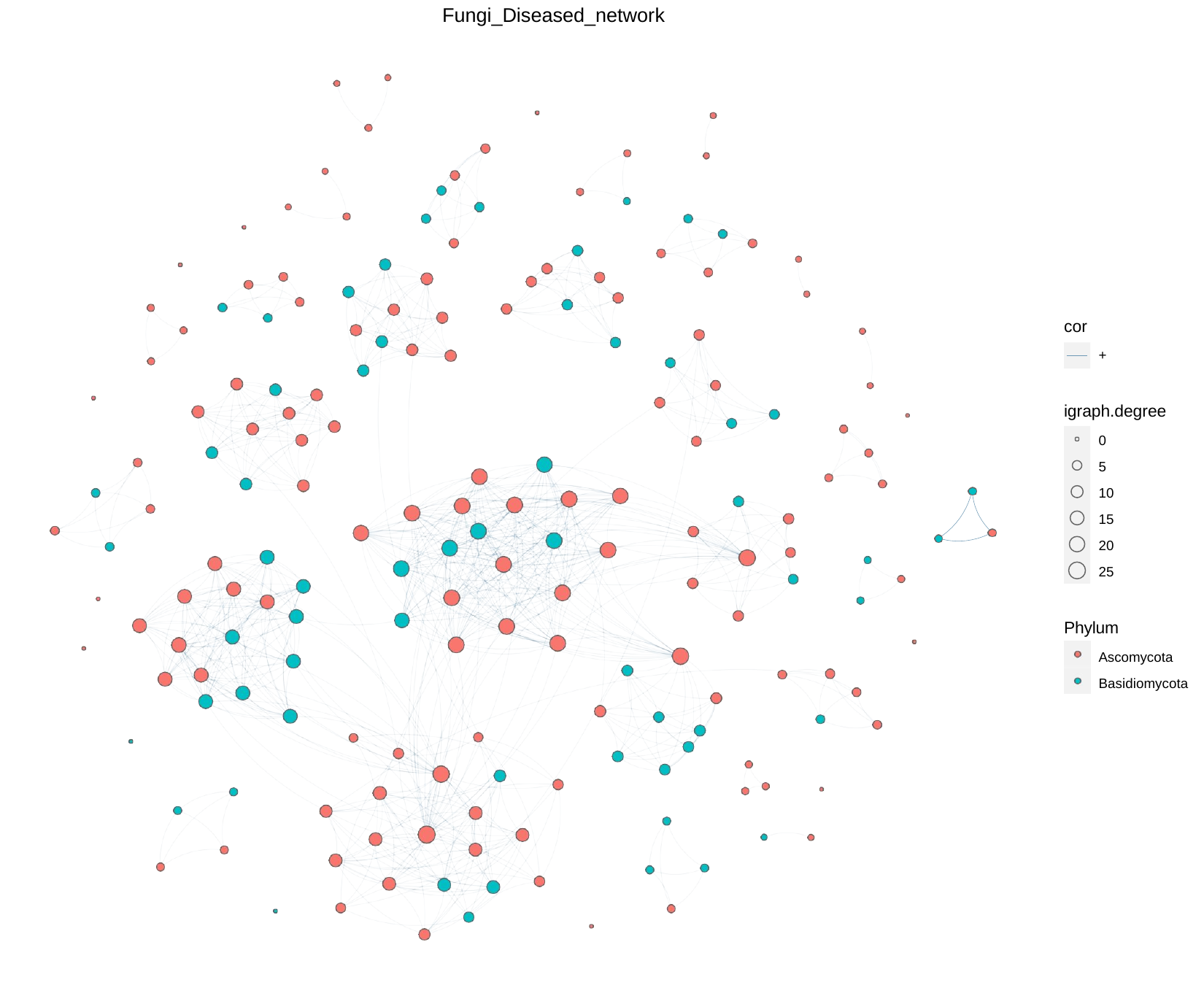

Fungi_Diseased_network
cor
+
igraph.degree
0
5
10
15
20
25
Phylum
Ascomycota Basidiomycota

## Slide 3
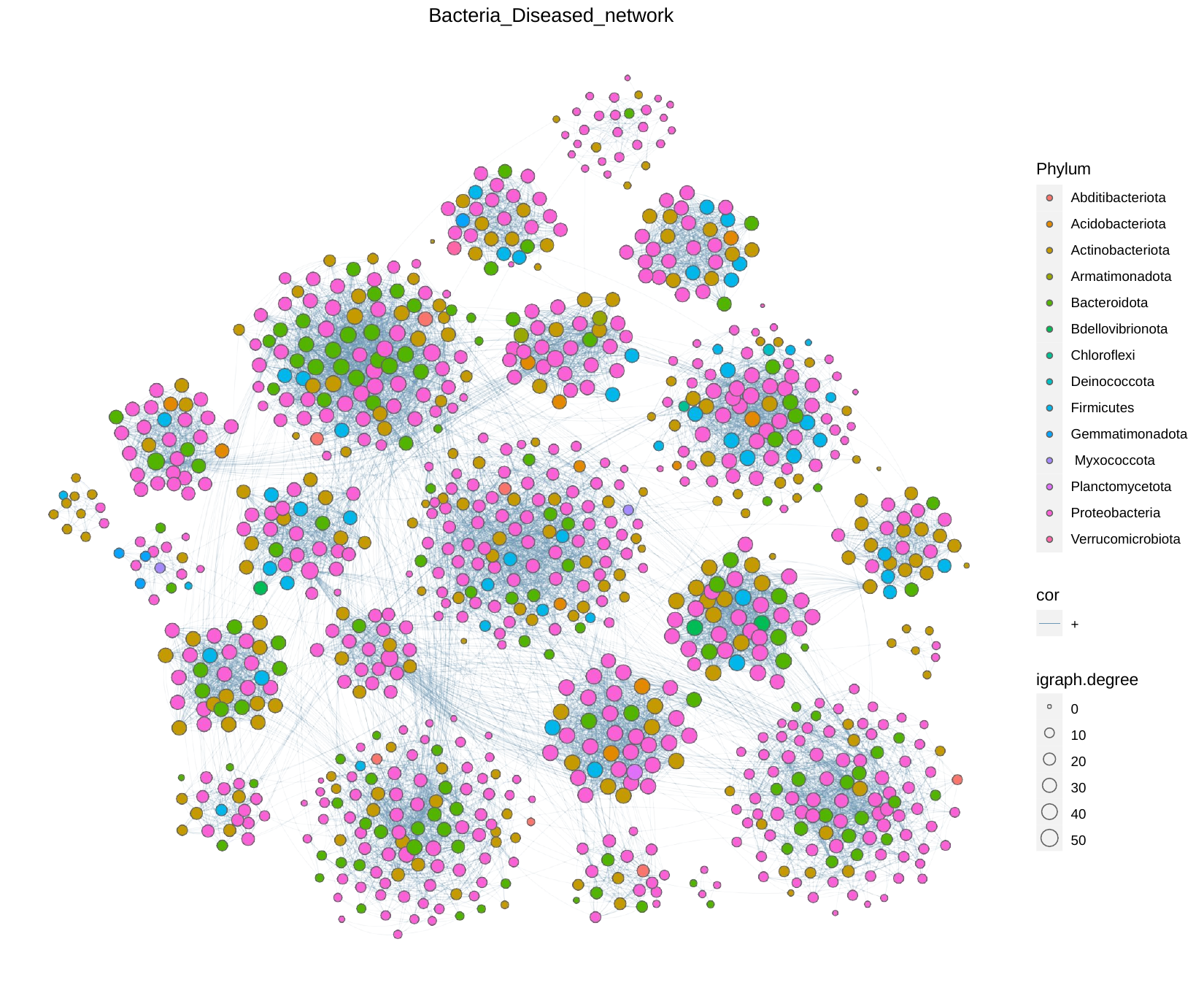

Bacteria_Diseased_network
Phylum
Abditibacteriota Acidobacteriota Actinobacteriota Armatimonadota Bacteroidota Bdellovibrionota Chloroflexi Deinococcota Firmicutes Gemmatimonadota Myxococcota Planctomycetota Proteobacteria Verrucomicrobiota
cor
+
igraph.degree
0
10
20
30
40
50

## Slide 4
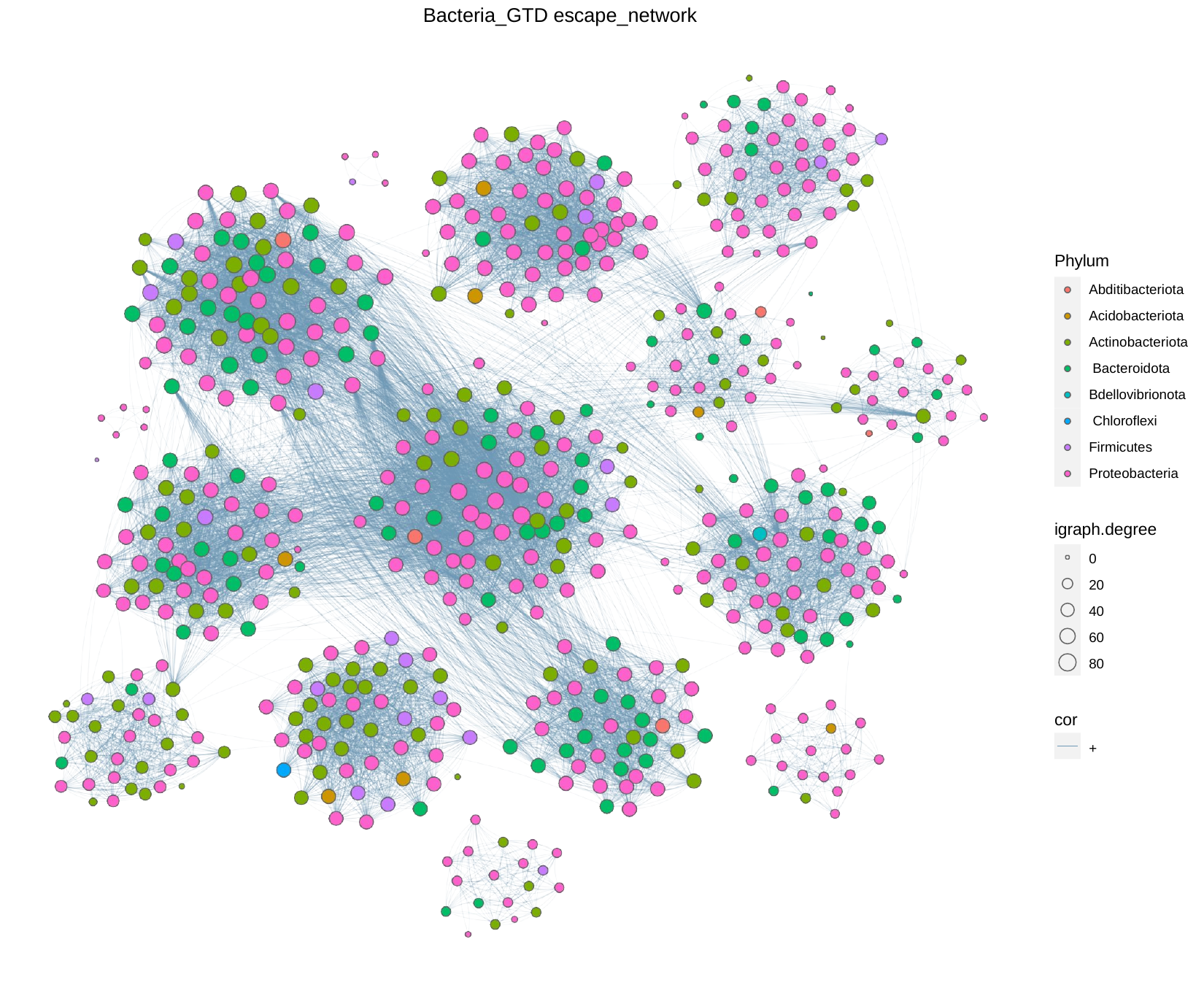

Bacteria_GTD escape_network
Phylum
Abditibacteriota Acidobacteriota Actinobacteriota Bacteroidota Bdellovibrionota Chloroflexi Firmicutes Proteobacteria
igraph.degree
0
20
40
60
80
cor
+
