# Supplementary material for: Grapevines escaping trunk diseases in New Zealand vineyards have a distinct microbiome structure
Source: Front Microbiol. 2023 Aug 23;14:1231832. doi: 10.3389/fmicb.2023.1231832 (PMC10482235; doi:10.3389/fmicb.2023.1231832)
Supplement: Supplementary file 1 [file Data_Sheet_1.zip › Supplementary_Material - Manuscript.pdf]

Supplementary Material

1.1 Supplementary Figures

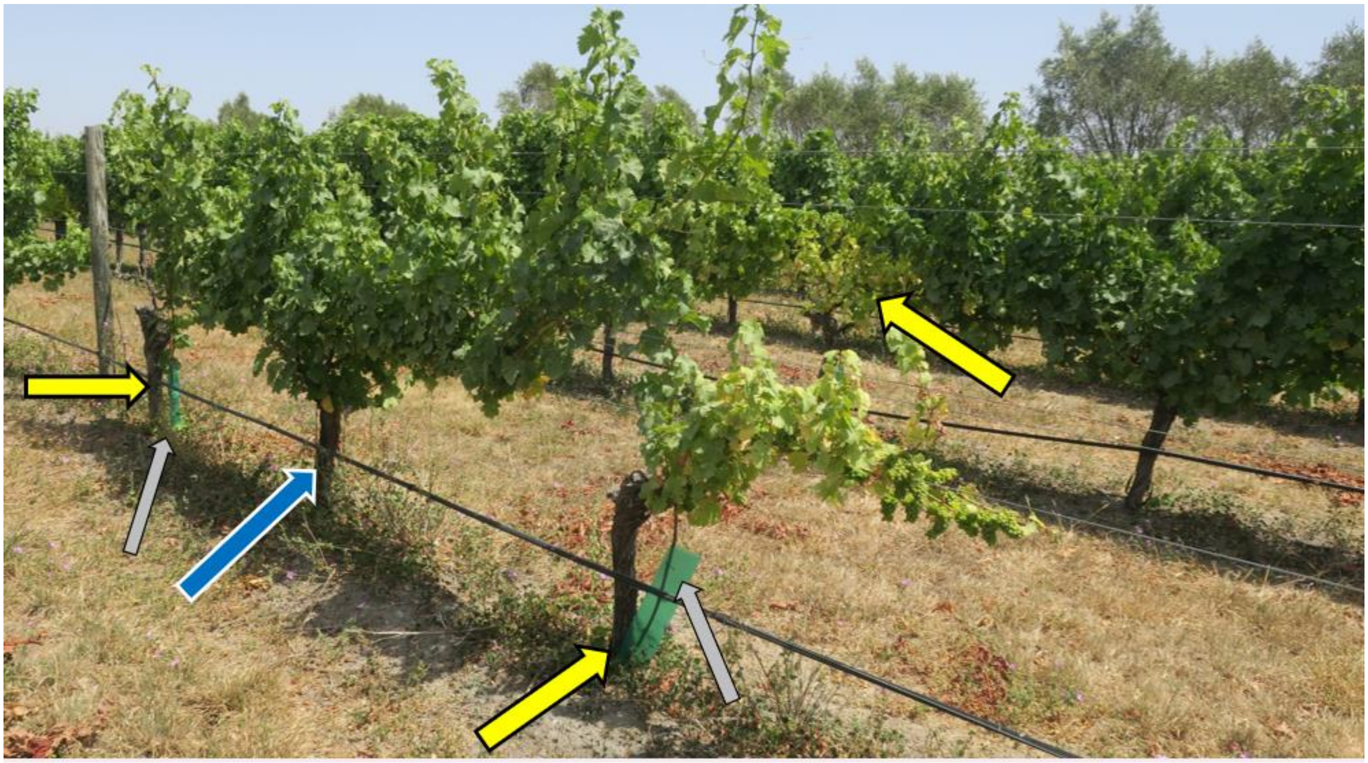

Supplementary Figure 1: A candidate grapevine trunk disease escape vine (blue arrow) surrounded by diseased vines (yellow arrows) and young water shoots (grey arrows) being trained to replace the diseased vines.

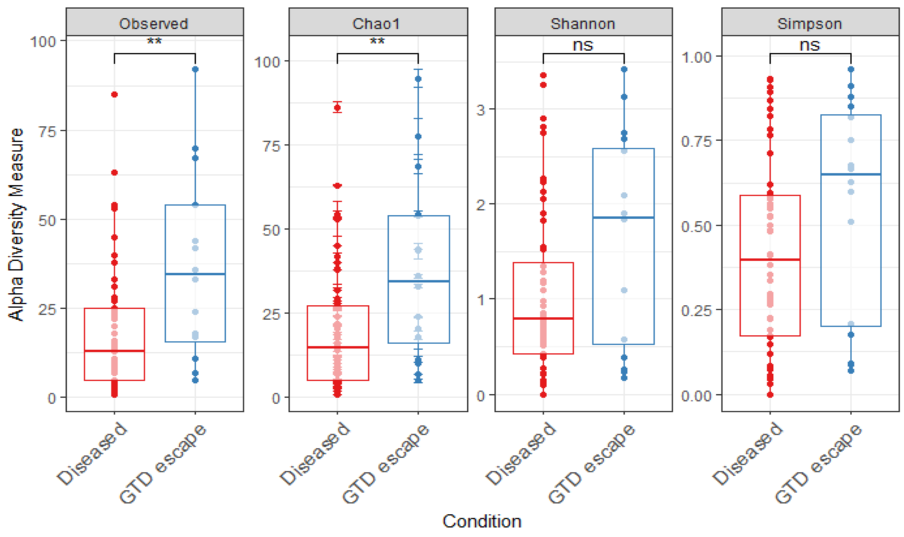

Supplementary Figure 2: Alpha diversity of fungal samples from woody trunk tissue of diseased and candidate GTD escape vine samples at the amplicon sequence variant level using Observed, Chao1, Shannon and Simpson indexes (** = p<0.02, ns= not significant)

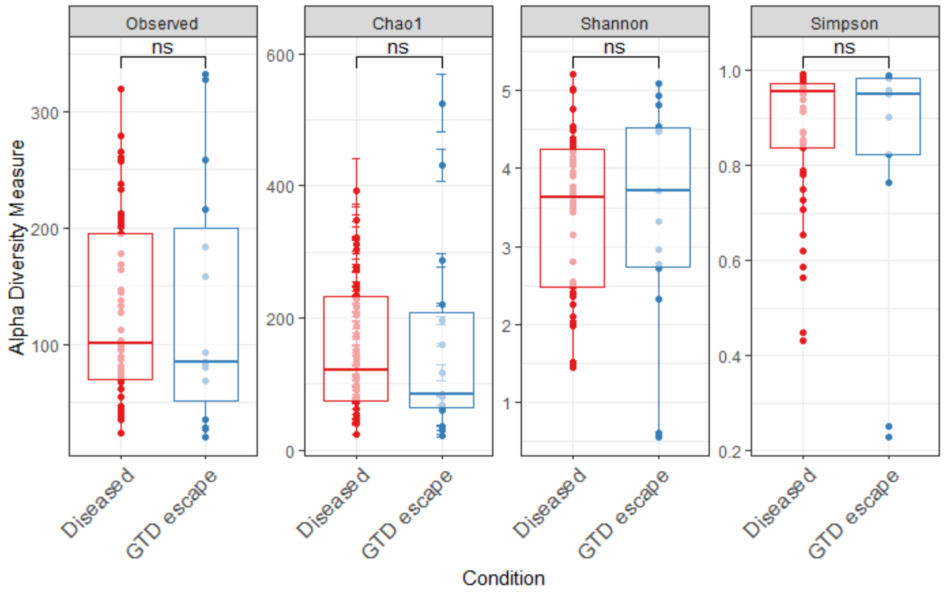

Supplementary Figure 3: Alpha diversity of bacterial samples from woody trunk tissue of diseased and candidate GTD escape vine samples at the amplicon sequence variant level using Observed, Chao1, Shannon and Simpson indexes (ns= not significant).

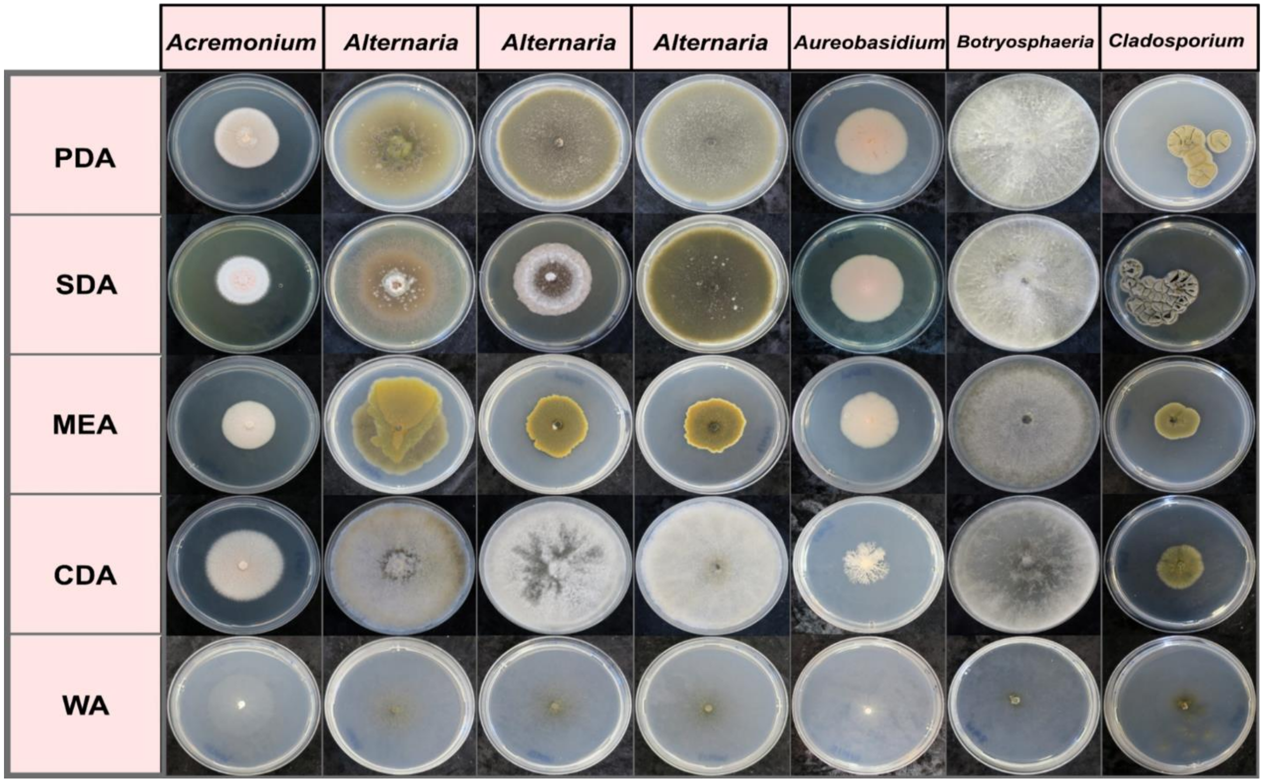

Supplementary Figure 4: The morphological structure of fungal genera isolated from grapevines and grown on different media for 10 days, 25°C dark (PDA: Potato Dextrose Agar, SDA: Sabouraud Dextrose Agar, MEA: Malt Extract Agar, CDA: Czapek-Dox Agar, WA: Water Agar)

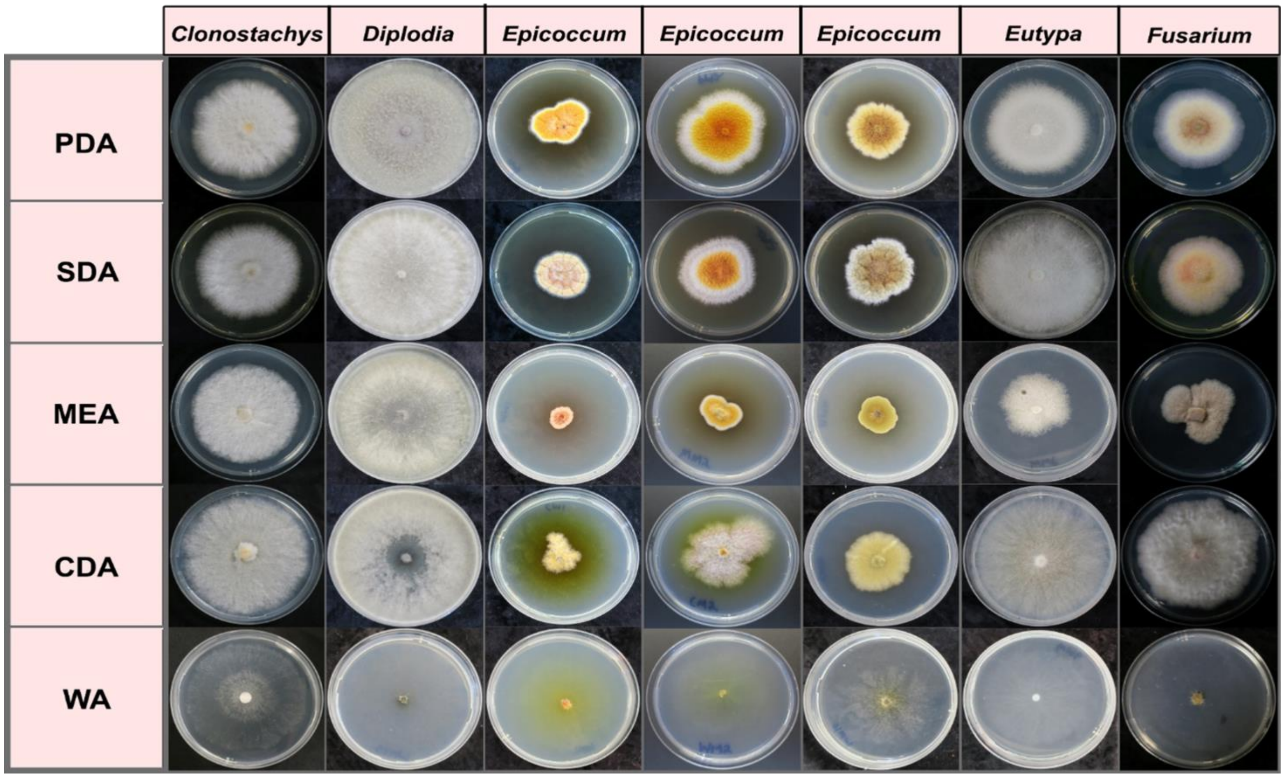

Supplementary Figure 5: The morphological structure of fungal genera isolated from grapevines and grown on different media for 10 days, 25ºC dark (PDA: Potato Dextrose Agar, SDA: Sabouraud Dextrose Agar, MEA: Malt Extract Agar, CDA: Czapek-Dox Agar, WA: Water Agar)

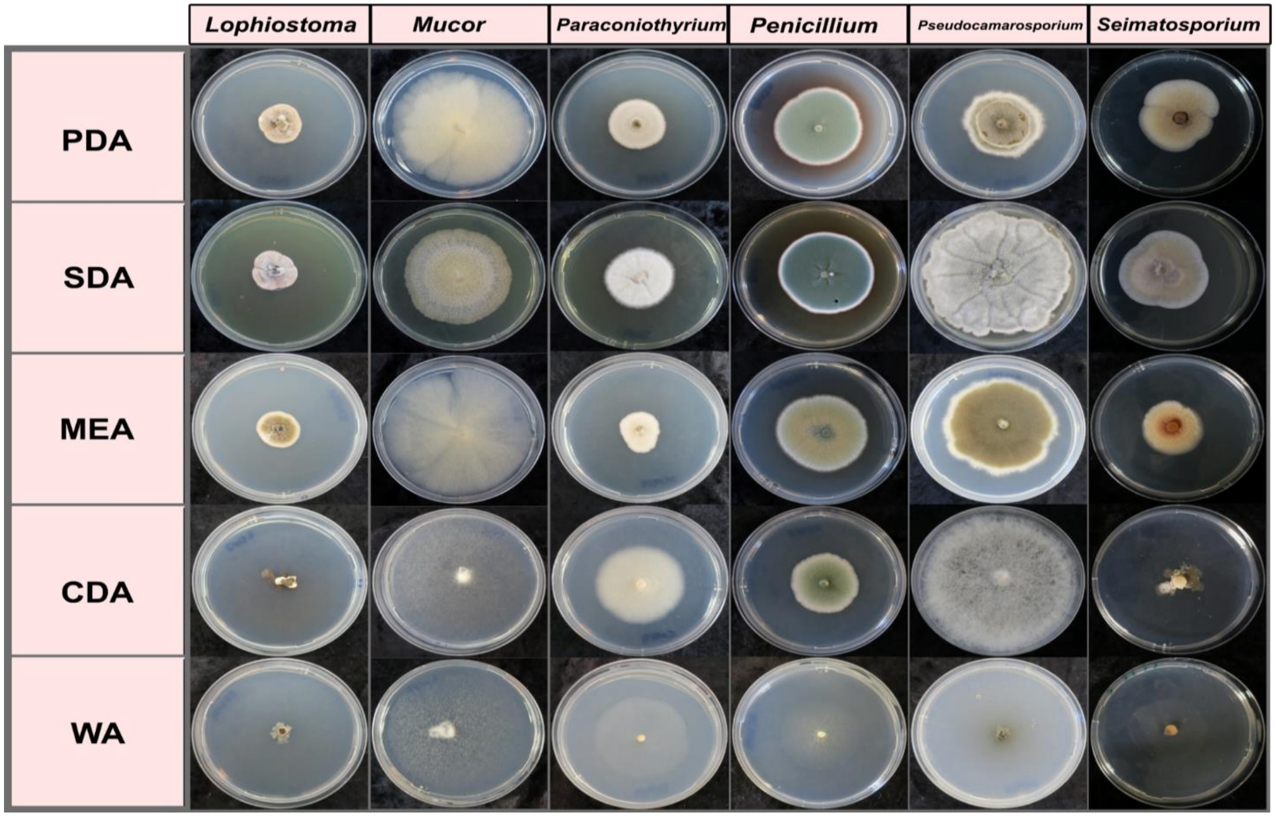

Supplementary Figure 6**:** The morphological structure of fungal genera isolated from grapevines and grown on different media for 10 days, 25ºC dark (PDA: Potato Dextrose Agar, SDA: Sabouraud Dextrose Agar, MEA: Malt Extract Agar, CDA: Czapek-Dox Agar, WA: Water Agar)

1.2 Supplementary Tables

Supplementary Table 1: Primers and adapter sequences used for DNA metabarcoding and microbial identification

Primer name	Sequence	Nextera adapter sequence
58A2R	AGTCCTGCGTTCTTCATCGAT	GTCTCGTGGGCTCGGAGATGTGTATAAGAGACAG
NSI1a	GATTGAATGGCTTAGTGAGK	TCGTCGGCAGCGTCAGATGTGTATAAGAGACAG
799F_MS	AACMGGATTAGATACCCKG	TCGTCGGCAGCGTCAGATGTGTATAAGAGACAG
1193R_MS	ACGTCATCCCCACCTTCC	GTCTCGTGGGCTCGGAGATGTGTATAAGAGACAG
ITS5	GGAAGTAAAAGTCGTAACAAGG	Not appplicable
ITS26	GCGGATCCATATGCTTAAGTTCAGCGGGT	Not appplicable
27F	AGAGTTTGATCCTGGTCAGAACGCT	Not appplicable
1492R	TACGGCTACCTTGTTACGACTTCACCCC	Not appplicable

Supplementary Table 2: Fungal isolates from GTD escape and diseased vines. Representative isolates were taken from each morphotype, and their ITS region was sequenced for molecular identification. Note: there were instances where different fungal genera were grouped into the same morphotype because they had similar morphological characteristics.

Morphotype	Organism	Escape	Diseased	Total
1.	*Epicoccum nigrum*	252	183	435
2.	*Epicoccum* c.f. *nigrum*	12	23	35
3.	*Cladosporium* sp	8	15	23
4.	*Pithomyces chartarum*	7	3	10
5.	*Fusarium* sp	1	16	17
6.	*Eutypa lata*	29	97	126
7.	*Clonostachys rosea*	4	25	29
8.	*Botryosphaeria, Neofusicoccum, Diplodia*	2	28	30
9.	*Mucor* sp	32	1	33
10.	*Epicoccum* c.f. *nigrum*	63	6	69
11.	*Alternaria*	52	81	133
12.	*Alternaria*	84	113	197
13.	*Diaporthe, Pithomyces*	0	5	5
14.	*Aureobasidium pullulans*	15	13	28
15.	*Seimatosporium vitis*	10	7	17
16.	*Ulocladium sp, Paraconiothyrium, Pseudocamarosporium* sp.	6	6	12
17.	*Penicillium*	5	3	8
18.	*Acremonium*	3	5	8
19.	*Lophiostoma, Pleosporales*	3	3	6
20.	Unidentified fungi	44	78	122
	Total	631	712	1343

Supplementary Table 3: Bacterial isolates from GTD escape and diseased grapevines. Out of the 151 isolates, 97 were identified to at least the genus level through the sequencing of their 16S rRNA gene region.

Genus	Escape	Diseased
Achromobacter	0	1
Acidovorax	1	2
Actinobacterium	2	0
Bacillus	1	3
Curtobacterium	9	6
Enterobacter	0	1
Erwinia	4	5
Frigoribacterium	0	1
Kocuria	3	0
Microbacterium	5	2
Micrococcus	2	1
Novosphingobium	0	3
Okibacterium	0	1
Pantoea	14	3
Plantibacter	1	1
Pseudomonas	13	5
Pseudoxanthomonas	0	1
Sanguibacter	2	1
Sphingobacterium	2	0
Sphingomonas	1	1
Xanthomonas	0	1
Unidentified	24	28
Total	84	67
